# Supplementary material for: Optogenetic dissection of descending behavioral control in Drosophila
Source: eLife. 2018 Jun 26;7:e34275. doi: 10.7554/eLife.34275 (PMC6031430; doi:10.7554/eLife.34275)
Supplement: Figure 2—source data 1. — The line by line analysis shown here follows the model of the selected examples described in Figure 2 of the main text. Also available at: http://www.biology.emory.edu/Berman/files/FigureS5_Cande_et_al.pdf. [file elife-34275-fig2-data1.zip › Stern_11-12-2017-RA-eLife-34275R1_Supplementary_File_1.pdf]

Figure S5.

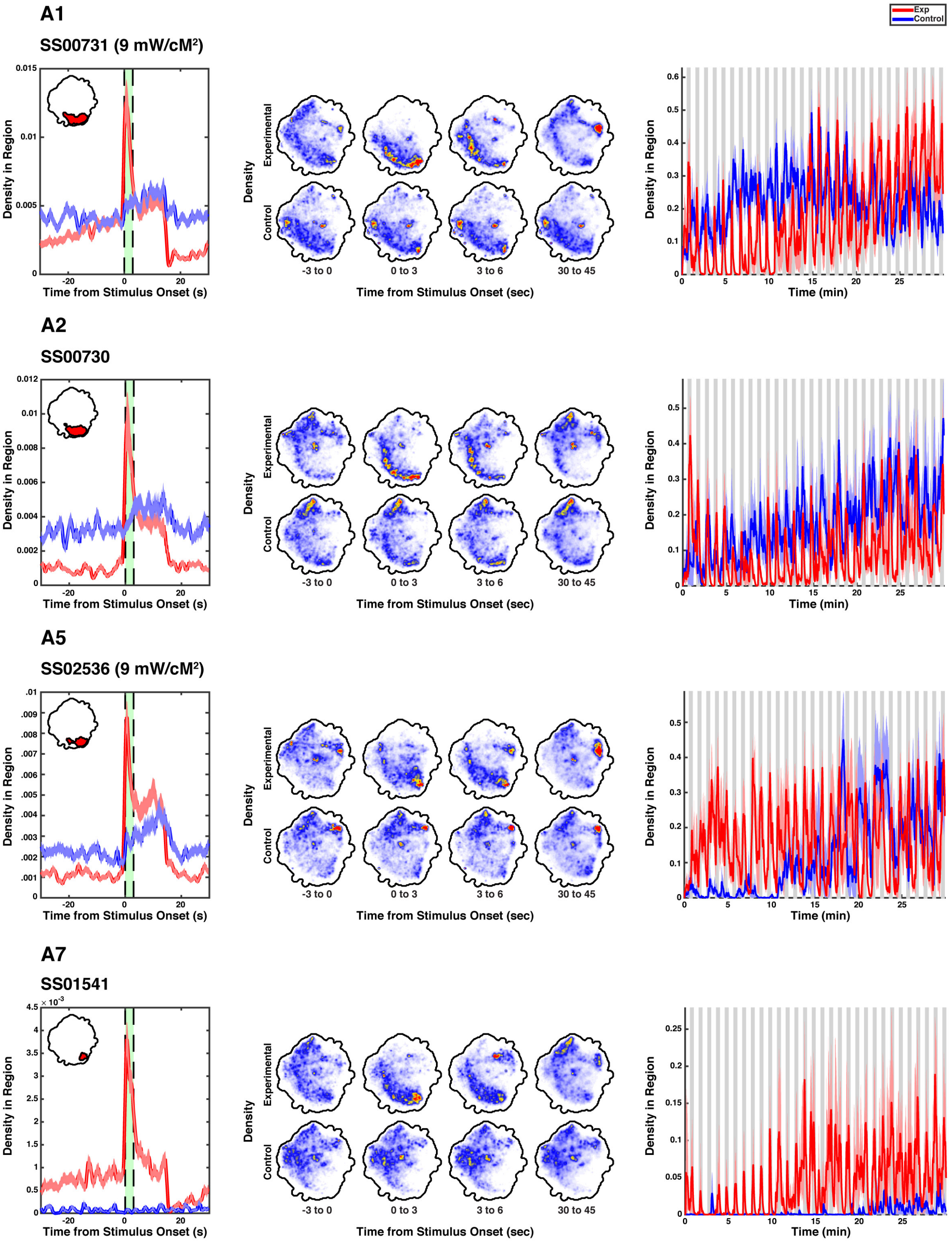
A1
SS00731 (9 mW/cM2)
Exp
Control
Density in Region
Time from Stimulus Onset (s)
Experimental
Control
Density
-3 to 0
0 to 3
3 to 6
30 to 45
Time from Stimulus Onset (sec)
Time (min)
A2
SS00730
A5
SS02536 (9 mW/cM2)
A7
SS01541

A7

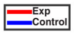

SS01542

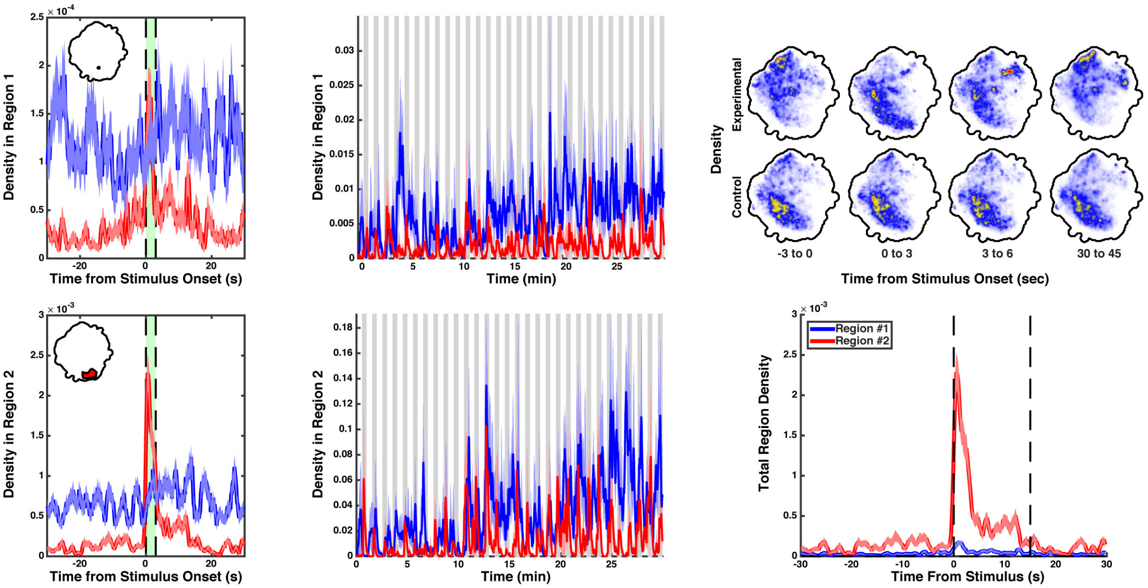

SS01560

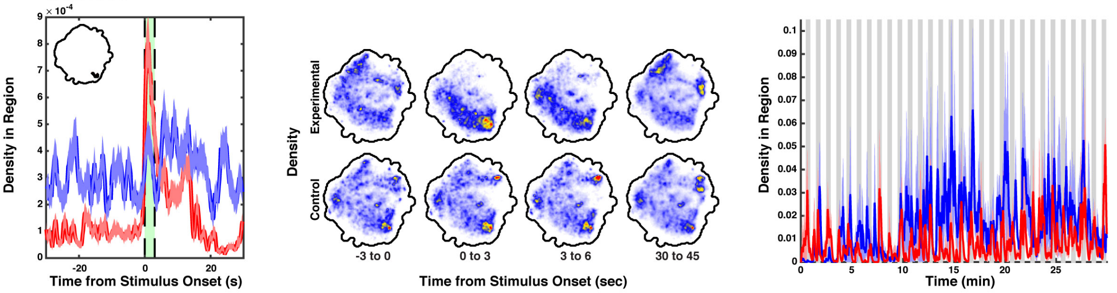

SS01571 (9mW/cM²)

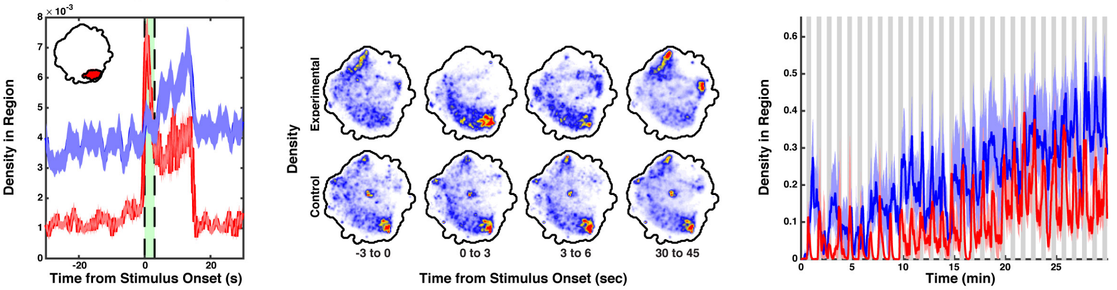

A8

SS02393 (9mW/cM²)

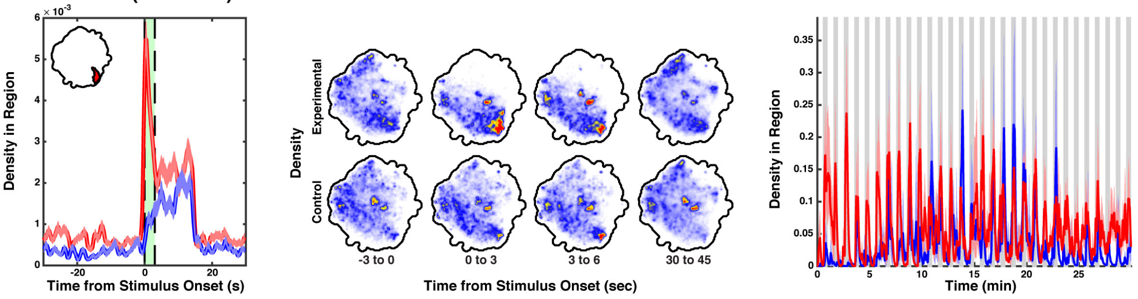

B1

SS02383 (9mW/cM²)

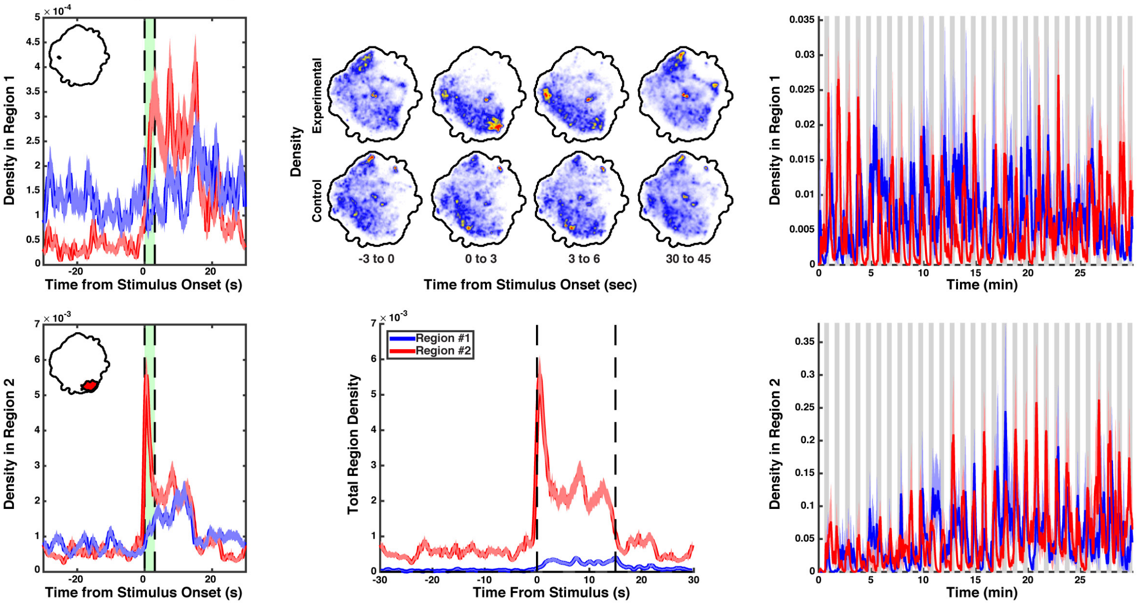

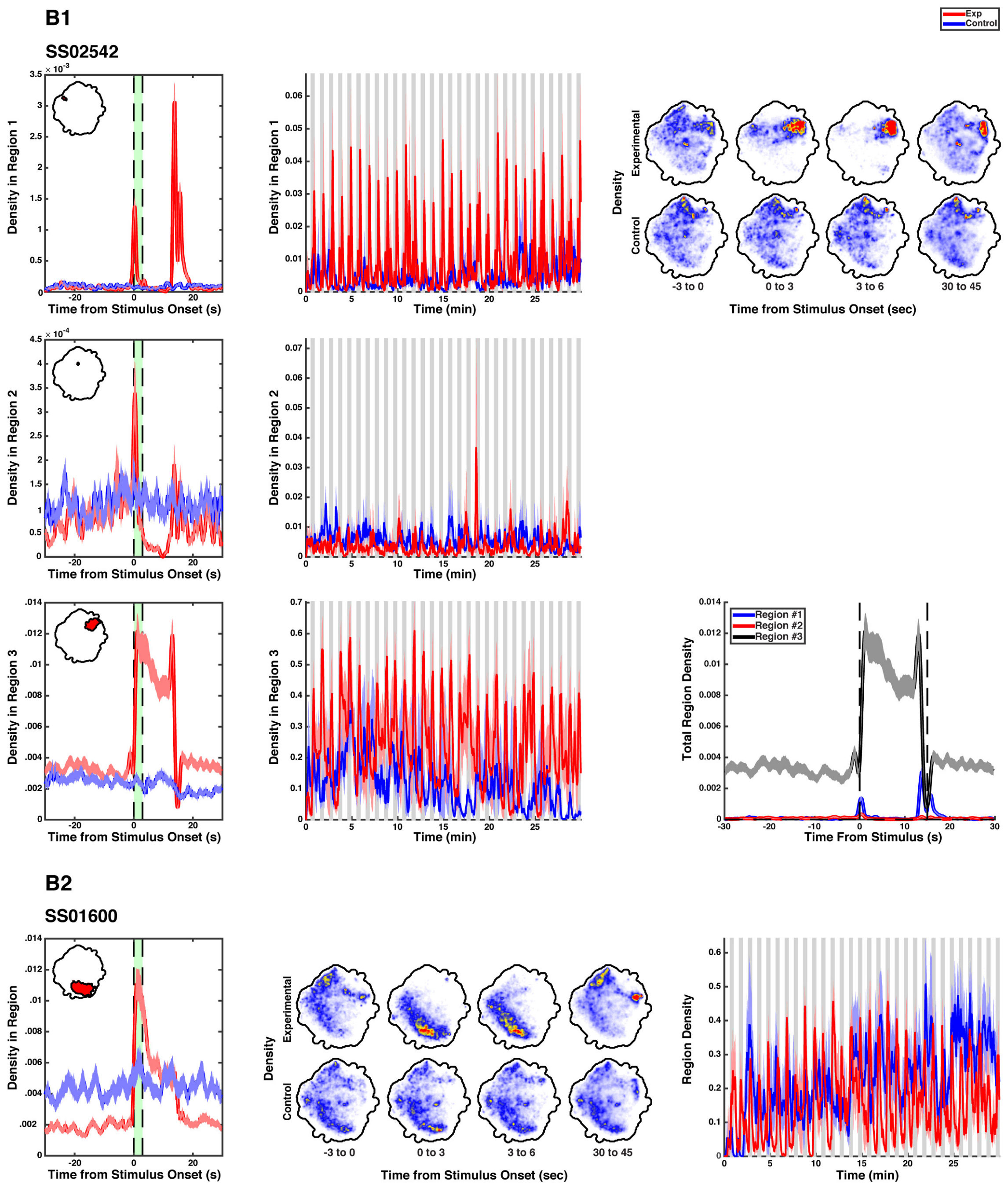
B1
SS02542
Exp
Control
Density in Region 1
Density in Region 2
Density in Region 3
Time from Stimulus Onset (s)
Time (min)
Density
Experimental
Control
-3 to 0
0 to 3
3 to 6
30 to 45
Time from Stimulus Onset (sec)
Region #1
Region #2
Region #3
Total Region Density
Time From Stimulus (s)
B2
SS01600
Density in Region
Region Density

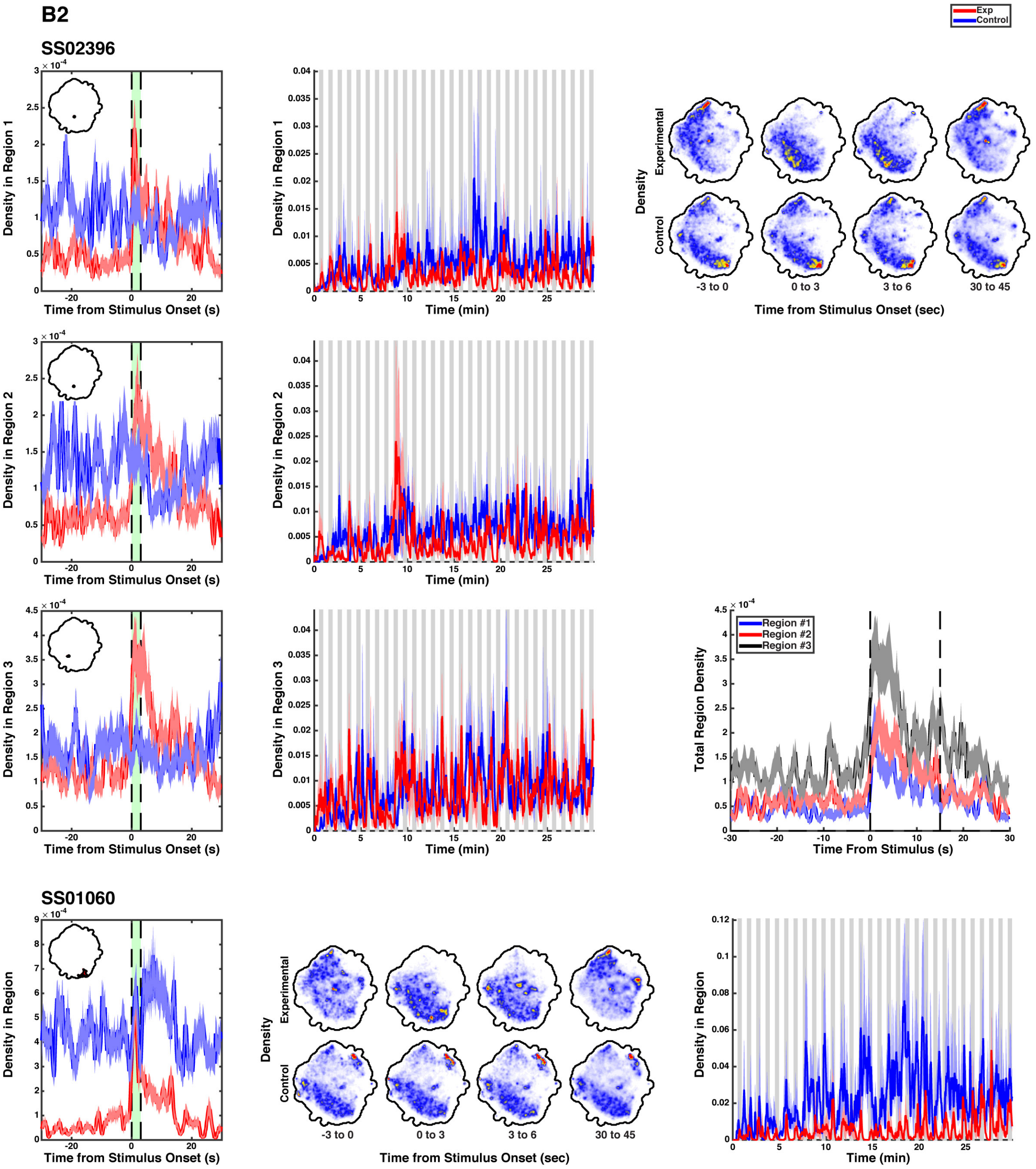
B2
Exp
Control
SS02396
Density in Region 1
Time from Stimulus Onset (s)
Density in Region 1
Time (min)
Experimental
Control
Density
-3 to 0
0 to 3
3 to 6
30 to 45
Time from Stimulus Onset (sec)
Density in Region 2
Time from Stimulus Onset (s)
Density in Region 2
Time (min)
Density in Region 3
Time from Stimulus Onset (s)
Density in Region 3
Time (min)
Region #1
Region #2
Region #3
Total Region Density
Time From Stimulus (s)
SS01060
Density in Region
Time from Stimulus Onset (s)
Experimental
Control
Density
-3 to 0
0 to 3
3 to 6
30 to 45
Time from Stimulus Onset (sec)
Density in Region
Time (min)

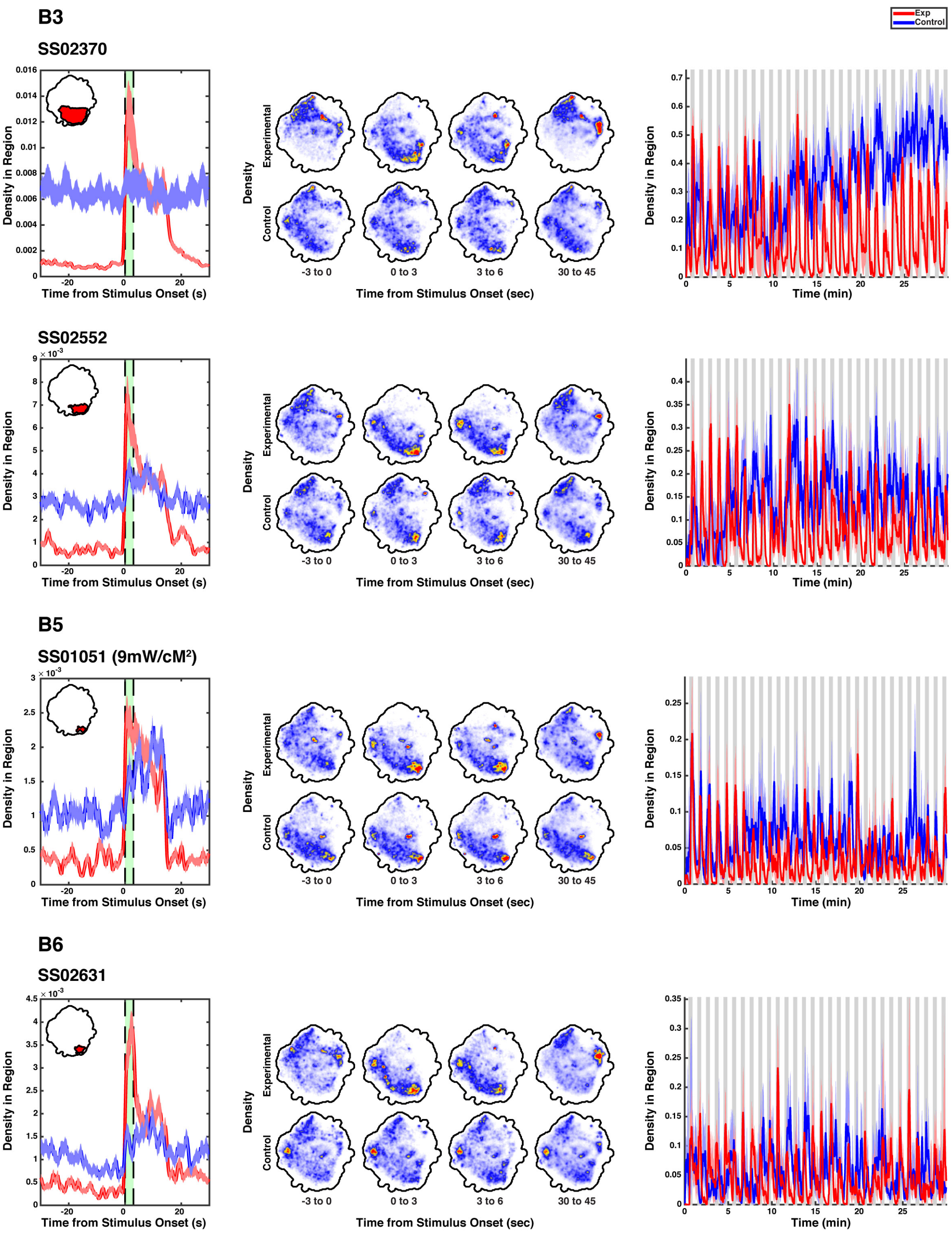

B3
SS02370
SS02552
B5
SS01051 (9mW/cM²)
B6
SS02631
Exp
Control
Density in Region
Time from Stimulus Onset (s)
Density
Experimental
Control
-3 to 0
0 to 3
3 to 6
30 to 45
Time from Stimulus Onset (sec)
Time (min)

C2

SS02395

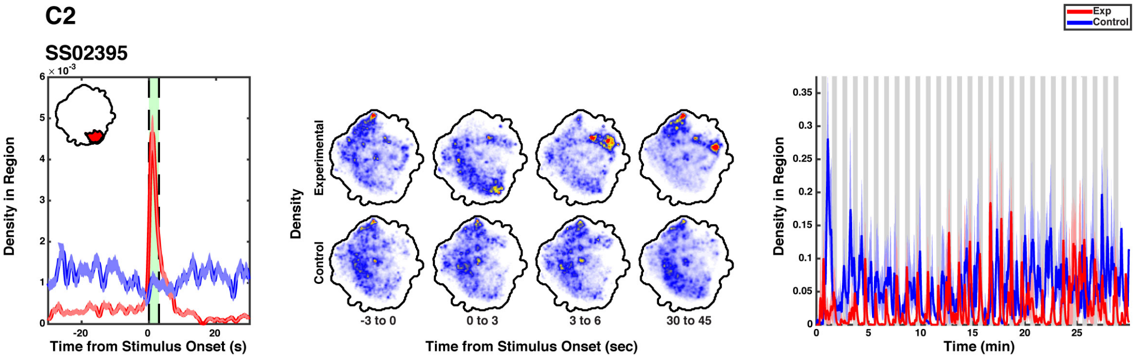

D2

SS01575

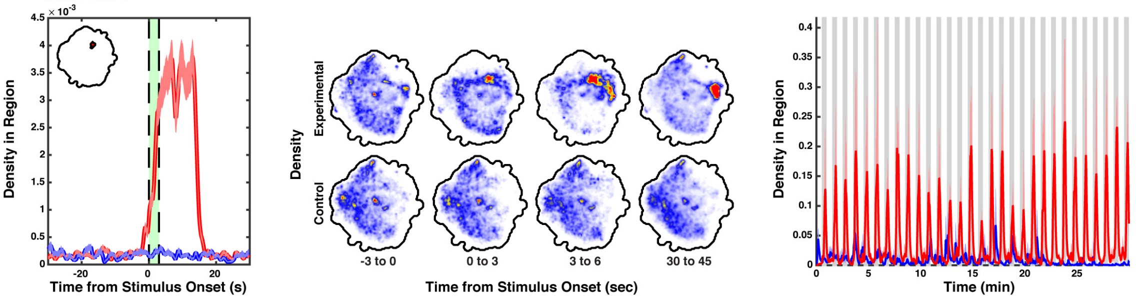

G1

SS01565

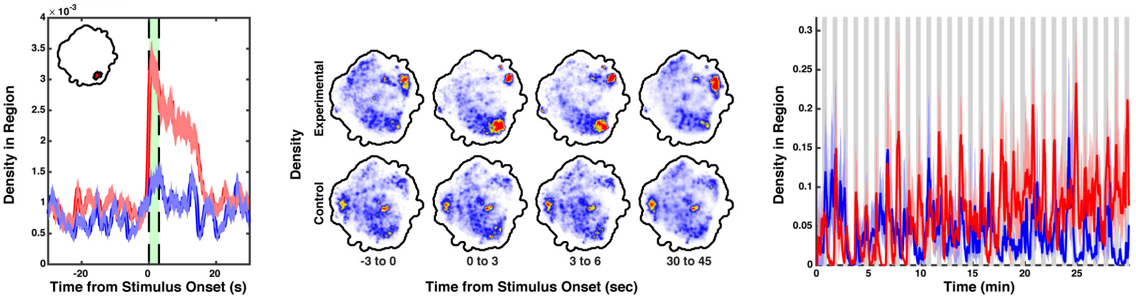

SS02275

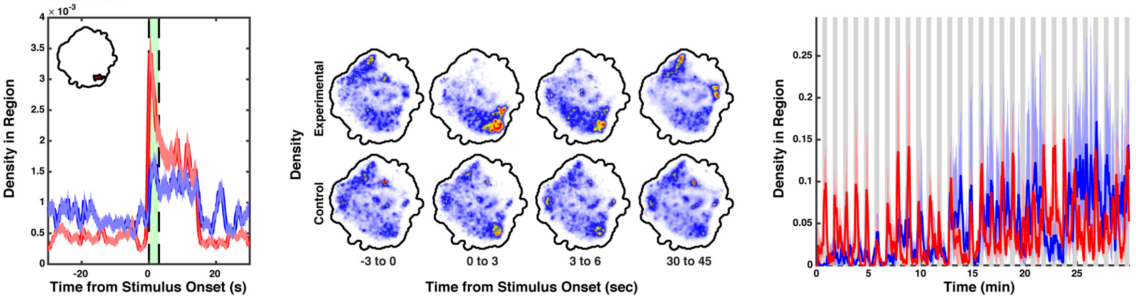

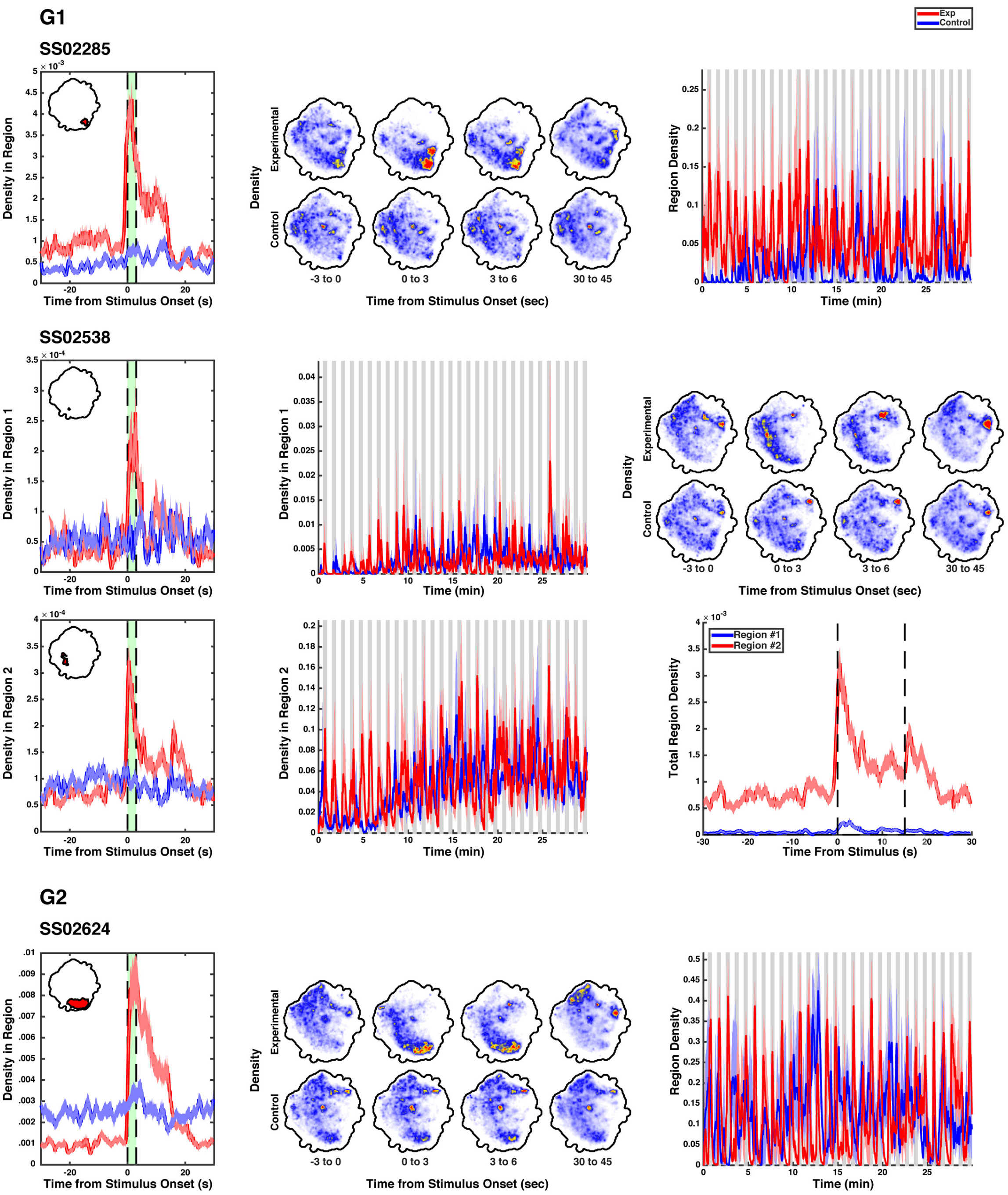
G1
SS02285
Exp
Control
Density in Region
Time from Stimulus Onset (s)
Experimental
Control
Density
-3 to 0
0 to 3
3 to 6
30 to 45
Time from Stimulus Onset (sec)
Region Density
Time (min)
SS02538
Density in Region 1
Density in Region 2
Region #1
Region #2
Total Region Density
Time From Stimulus (s)
G2
SS02624

G2 - SS01561

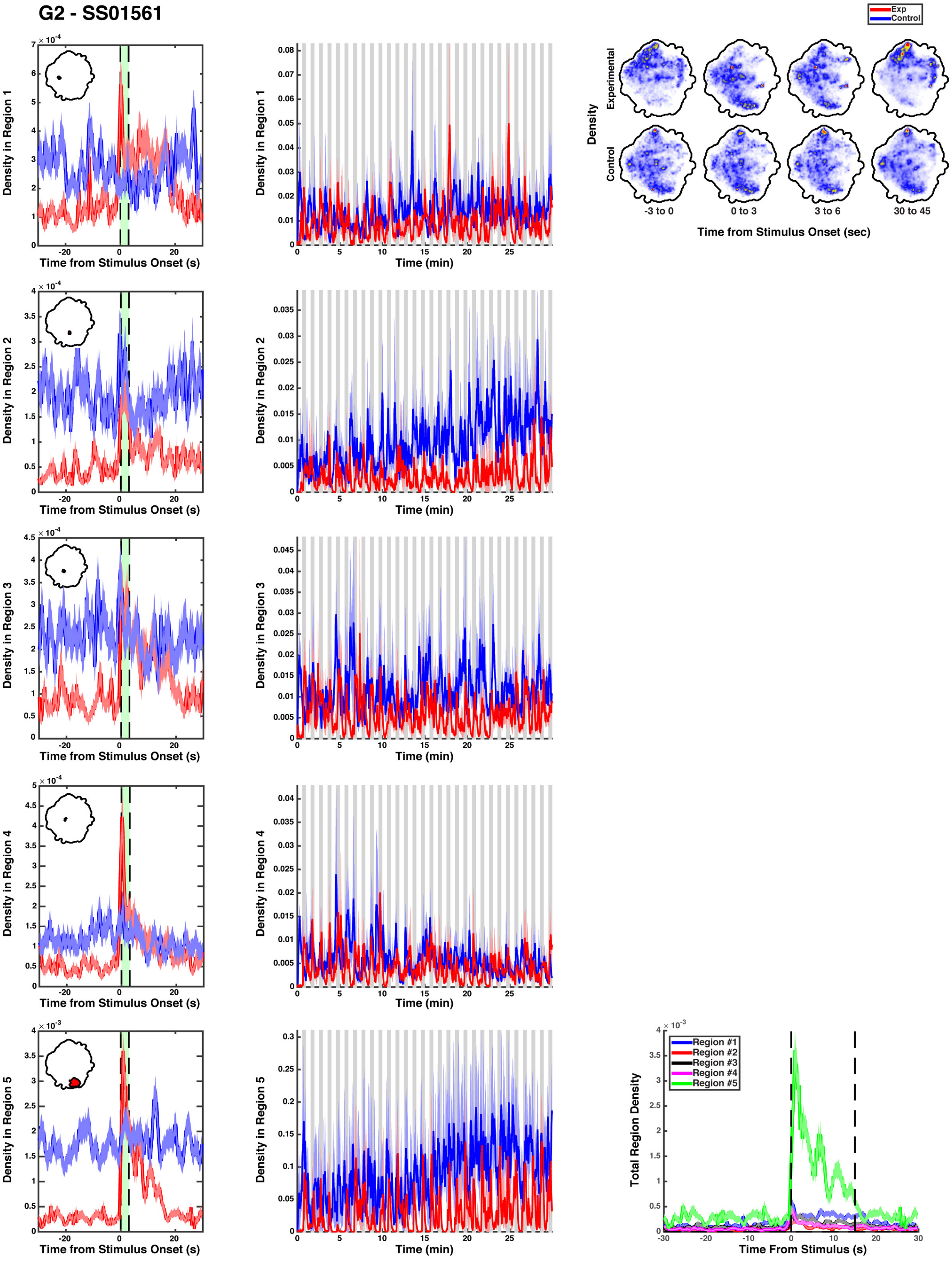
Exp
Control
Density in Region 1
Density in Region 2
Density in Region 3
Density in Region 4
Density in Region 5
Time from Stimulus Onset (s)
Time (min)
Density
Experimental
Control
-3 to 0
0 to 3
3 to 6
30 to 45
Time from Stimulus Onset (sec)
Region #1
Region #2
Region #3
Region #4
Region #5
Total Region Density
Time From Stimulus (s)

G2

SS01073 (9mW/cM²)

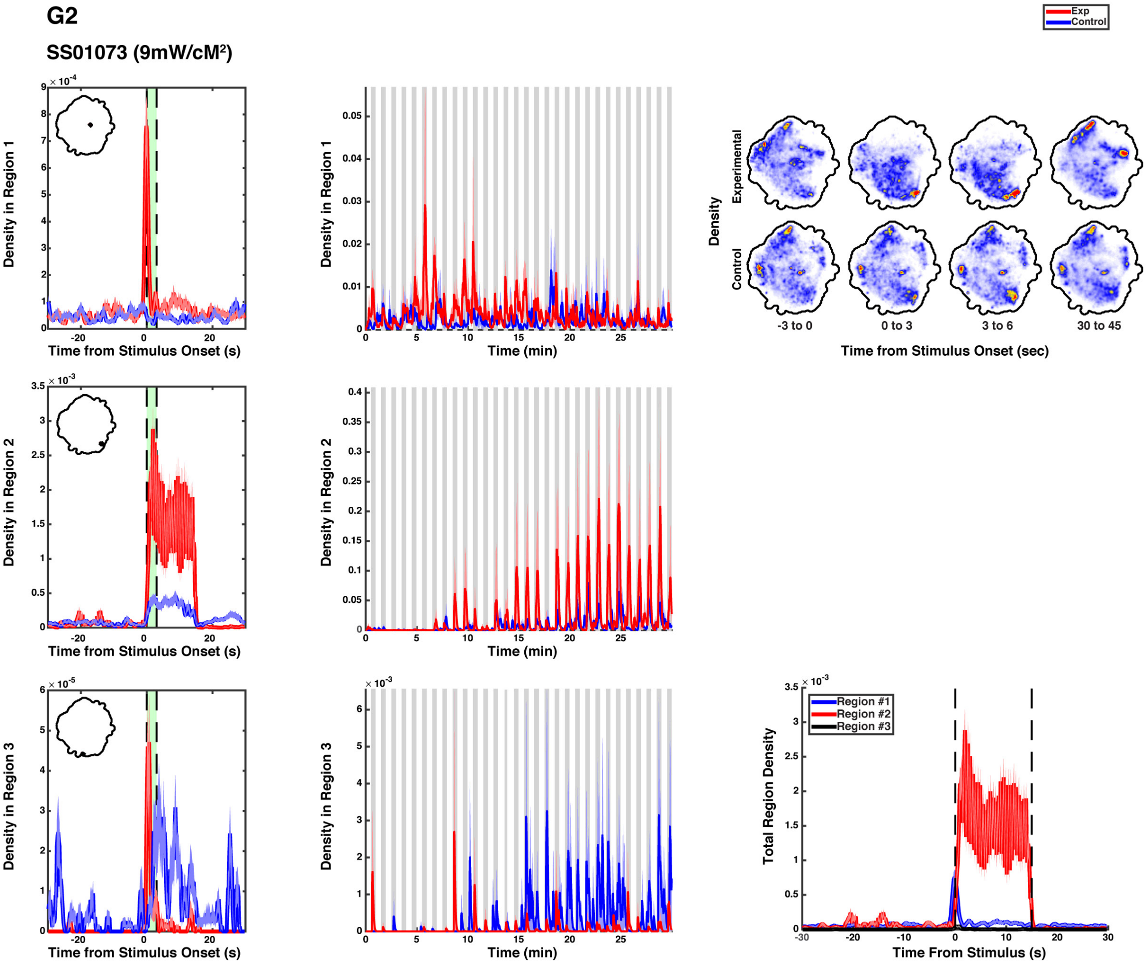

SS02634

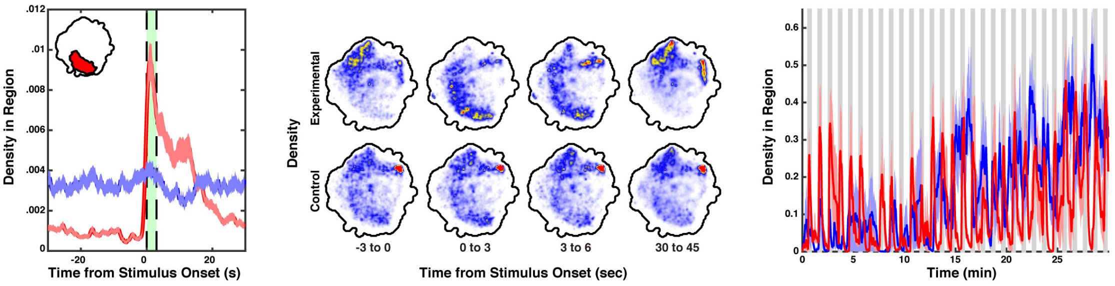

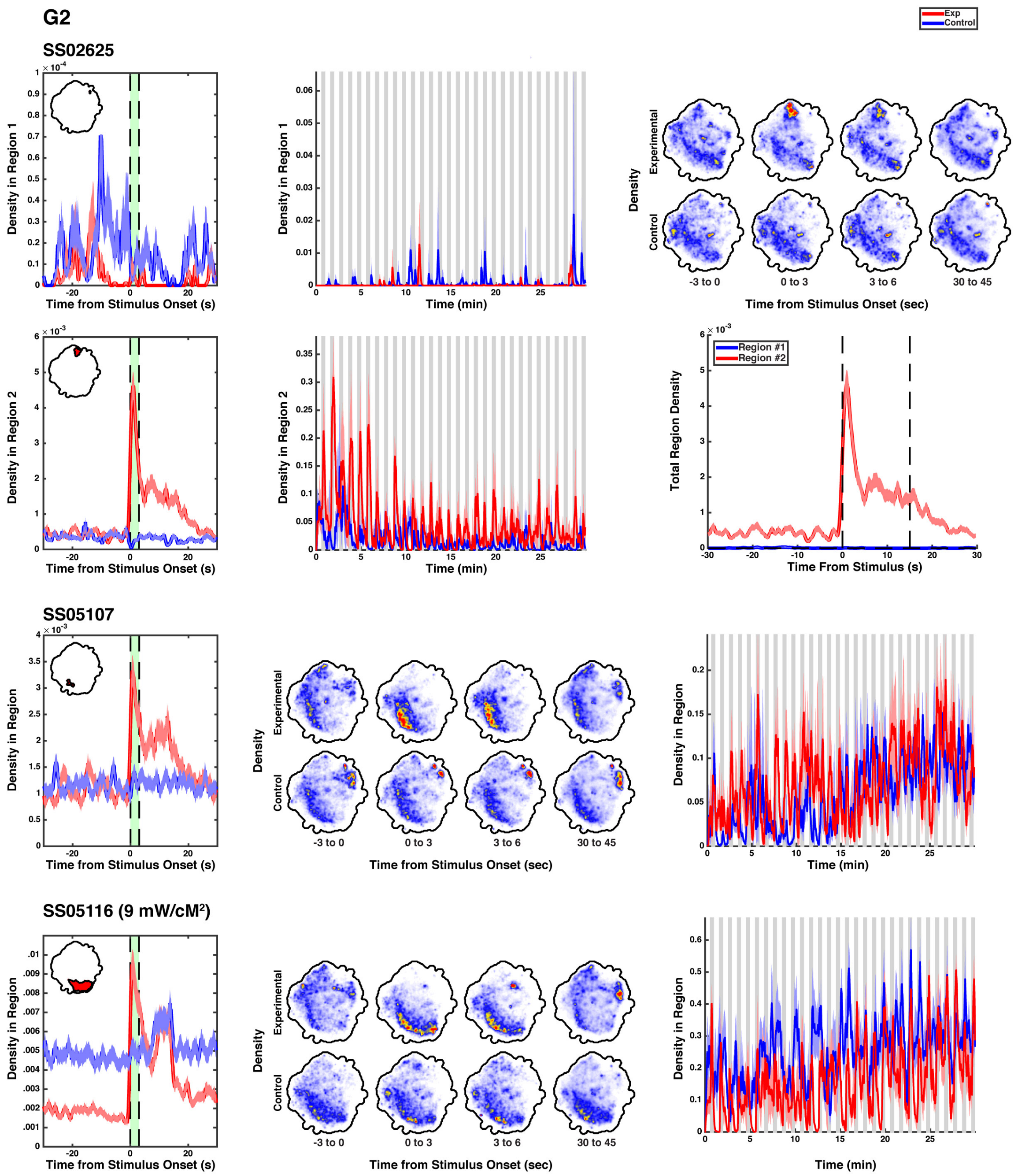
G2
Exp
Control
SS02625
Density in Region 1
Time from Stimulus Onset (s)
Time (min)
Experimental
Control
Density
-3 to 0
0 to 3
3 to 6
30 to 45
Time from Stimulus Onset (sec)
Density in Region 2
Region #1
Region #2
Total Region Density
Time From Stimulus (s)
SS05107
Density in Region
SS05116 (9 mW/cM²)

G3

SS02548

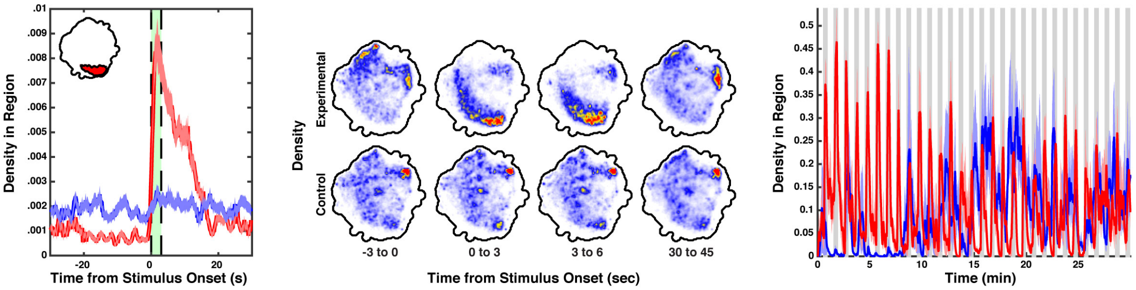

SS02633

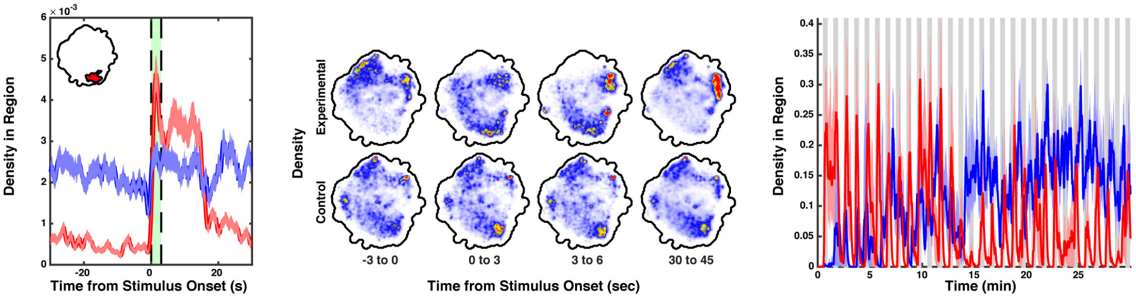

G3

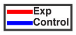

SS05098 (9 mW/cM^2)

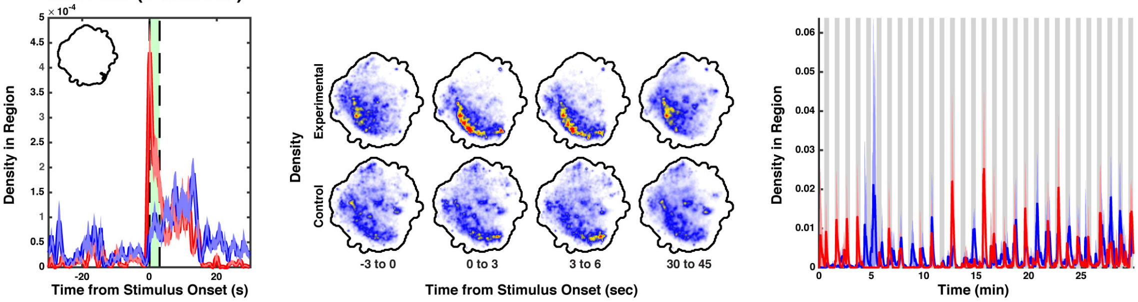

SS05108 (9 mW/cM^2)

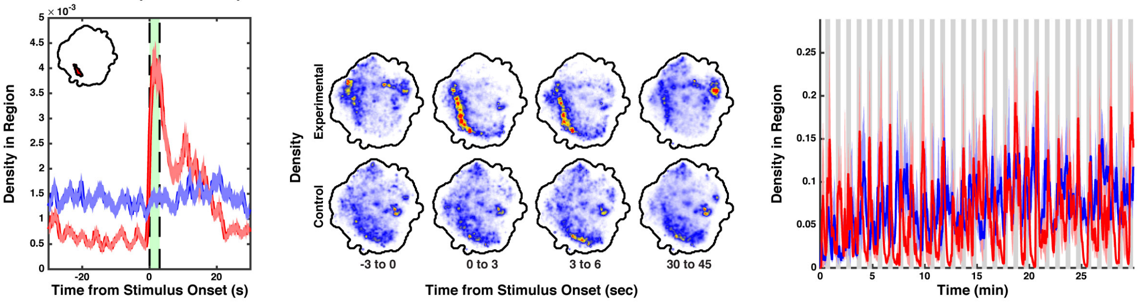

G7

SS01074

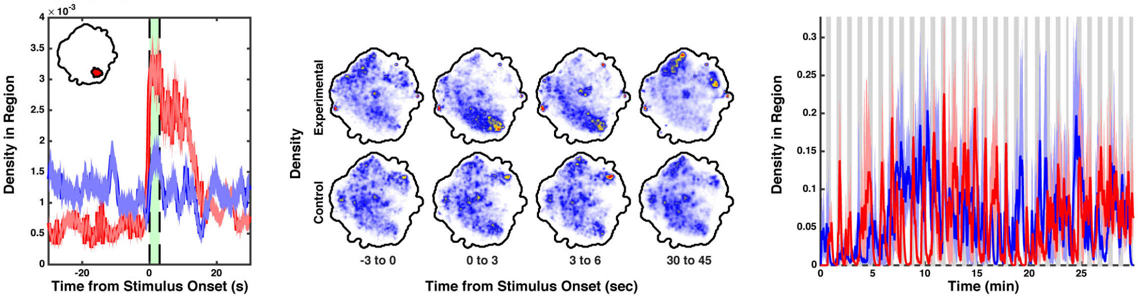

SS01597

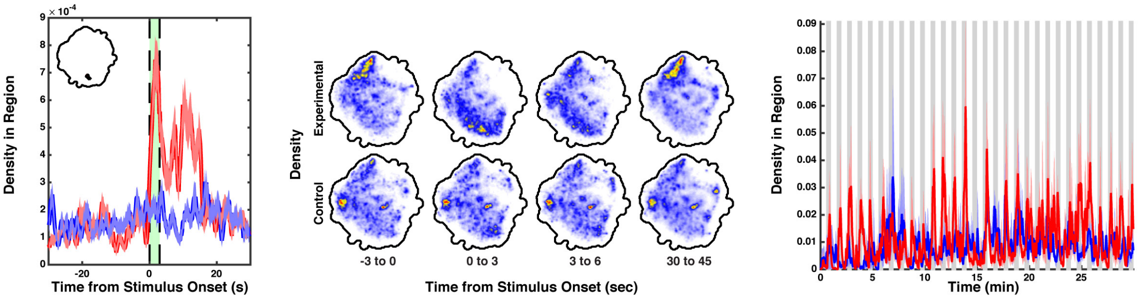

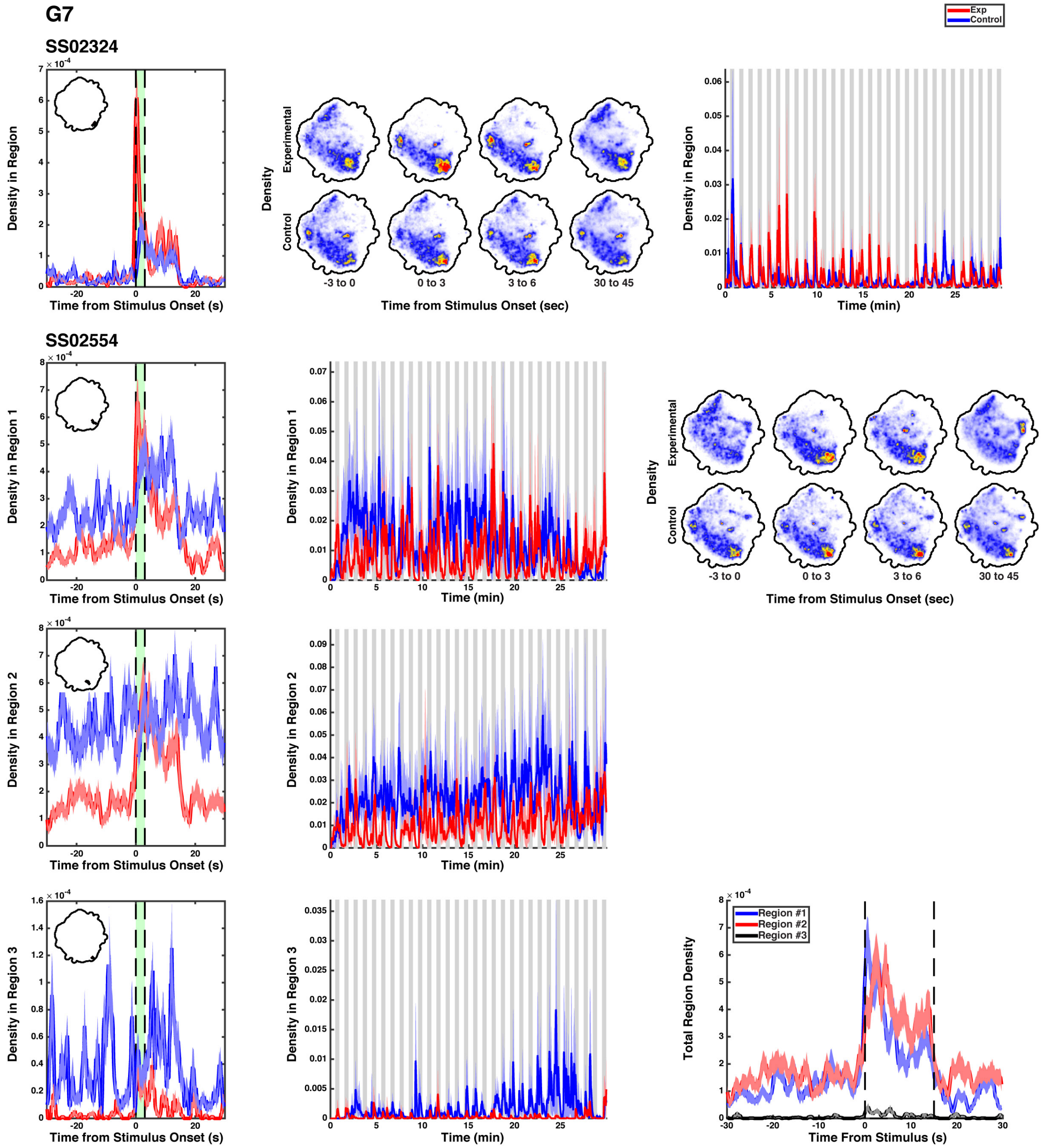
G7
SS02324
SS02554
Exp
Control
Density in Region
Density in Region 1
Density in Region 2
Density in Region 3
Time from Stimulus Onset (s)
Experimental
Control
Density
-3 to 0
0 to 3
3 to 6
30 to 45
Time from Stimulus Onset (sec)
Time (min)
Region #1
Region #2
Region #3
Total Region Density
Time From Stimulus (s)

G10

SS01547

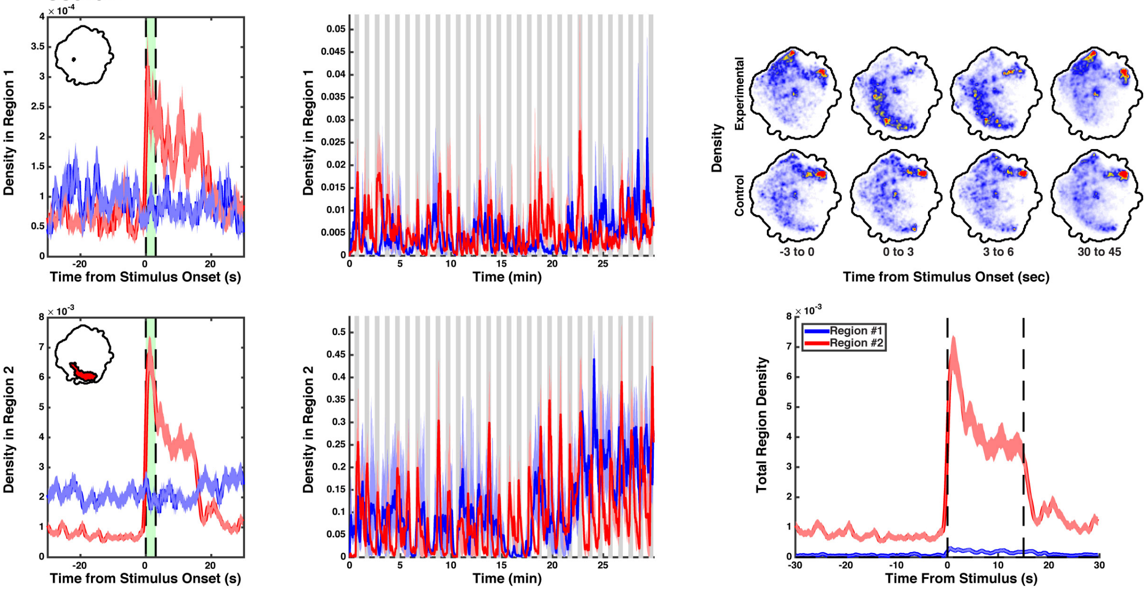

SS02111

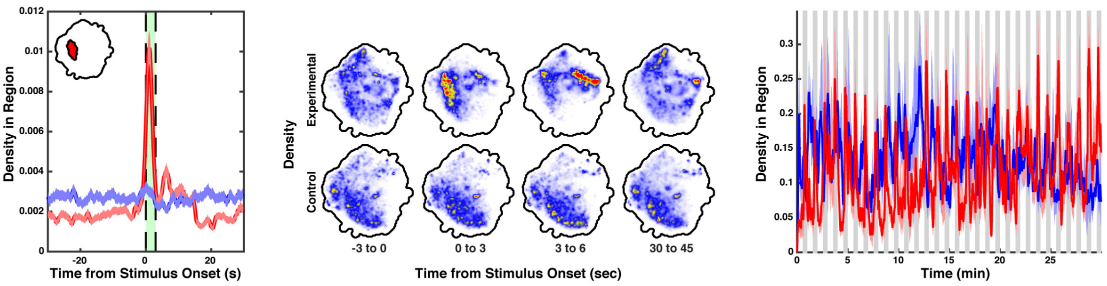

G10

SS02278

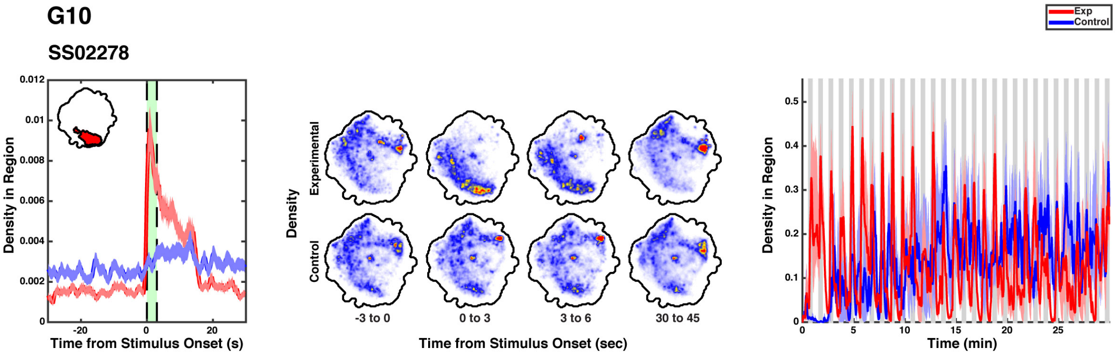

G11

SS01550

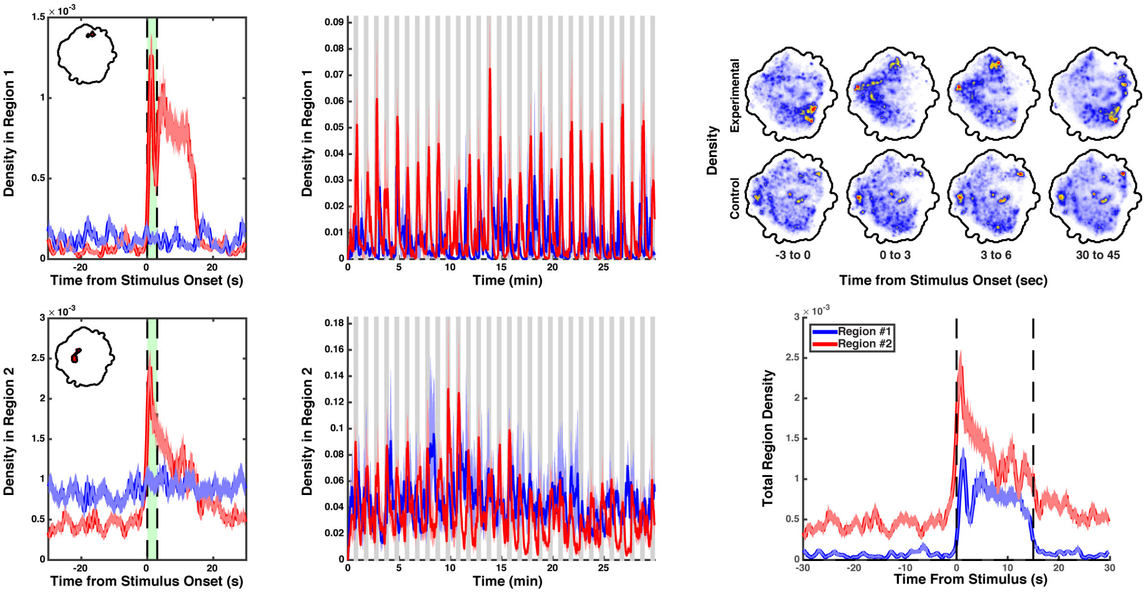

SS01566

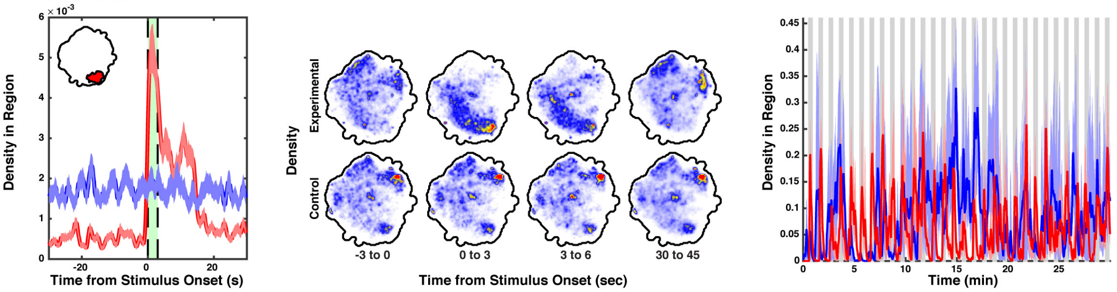

G11

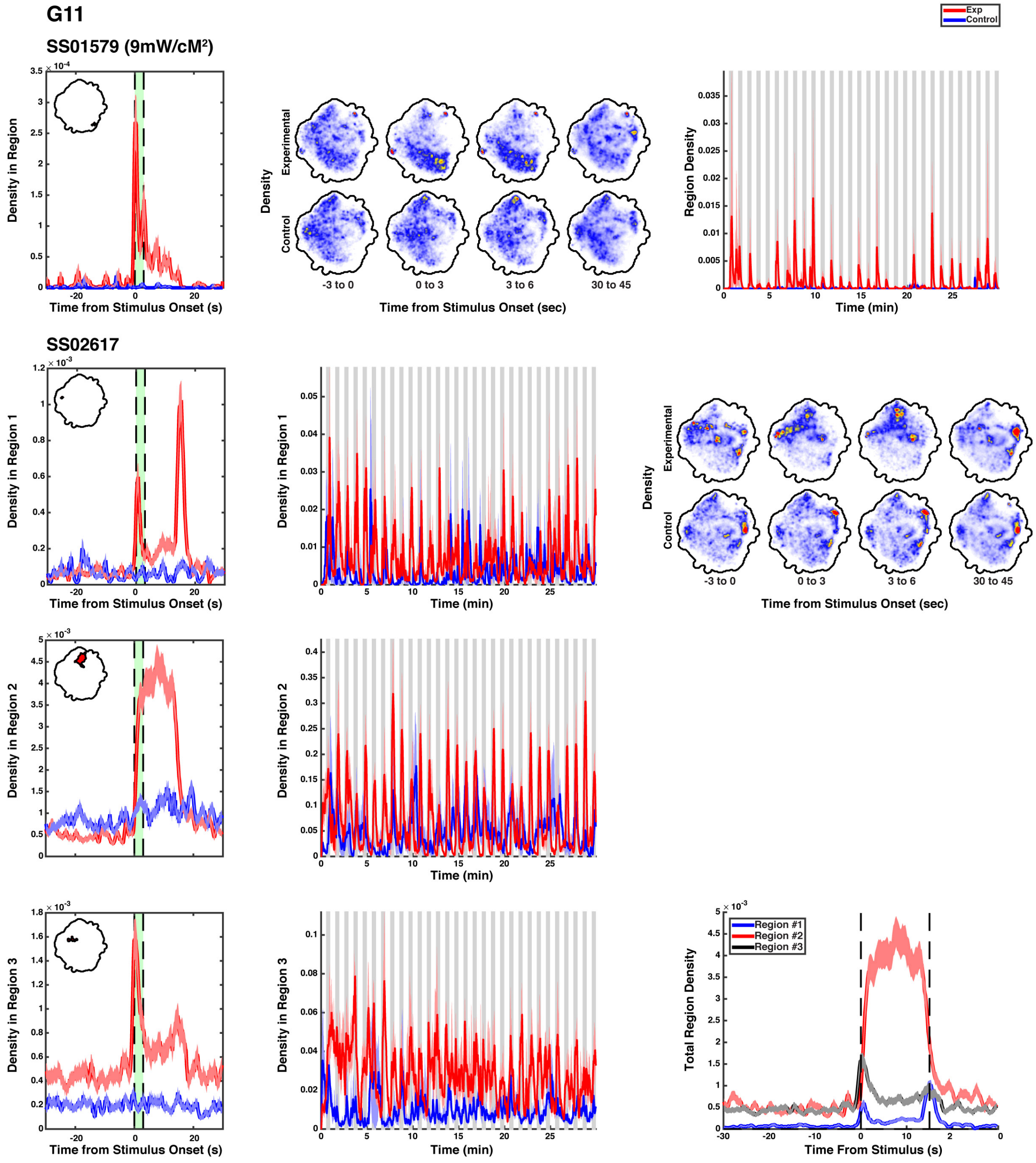

Exp
Control
SS01579 (9mW/cM²)
Density in Region
Time from Stimulus Onset (s)
Density
Experimental
Control
-3 to 0
0 to 3
3 to 6
30 to 45
Time from Stimulus Onset (sec)
Region Density
Time (min)
SS02617
Density in Region 1
Density in Region 2
Density in Region 3
Region #1
Region #2
Region #3
Total Region Density
Time From Stimulus (s)

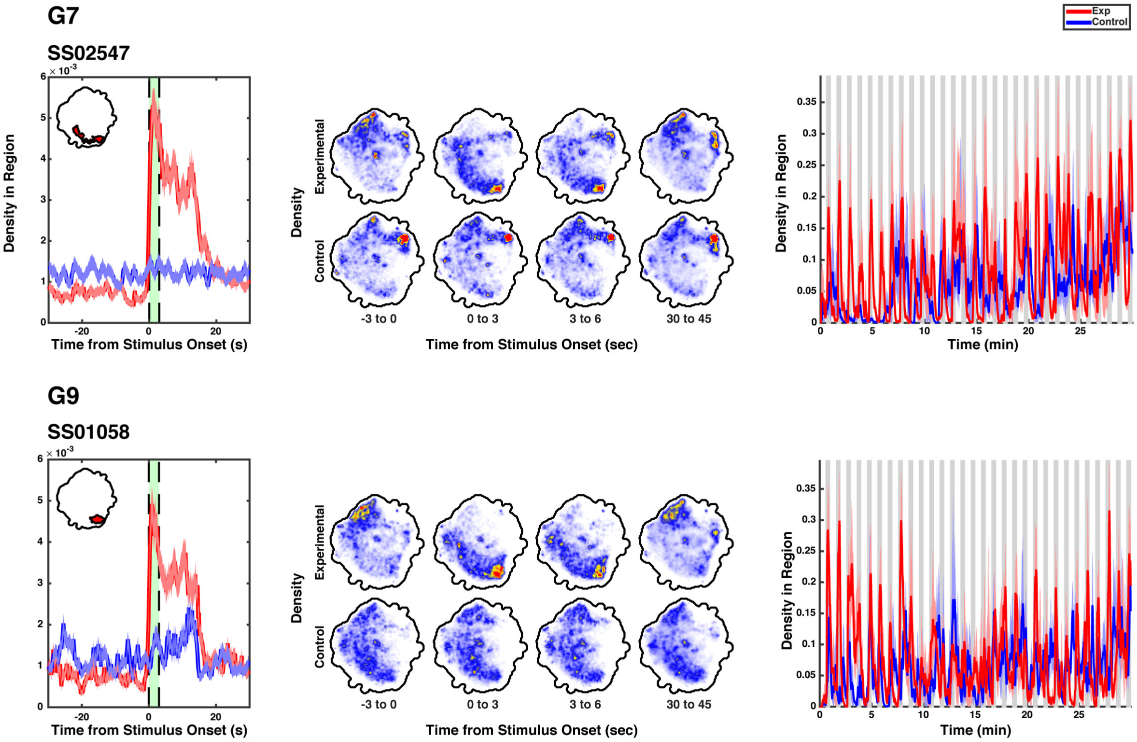

G7
SS02547
Exp
Control
Density in Region
Time from Stimulus Onset (s)
Density
Experimental
Control
-3 to 0
0 to 3
3 to 6
30 to 45
Time from Stimulus Onset (sec)
Density in Region
Time (min)
G9
SS01058
Density in Region
Time from Stimulus Onset (s)
Density
Experimental
Control
-3 to 0
0 to 3
3 to 6
30 to 45
Time from Stimulus Onset (sec)
Density in Region
Time (min)

G11

SS02391

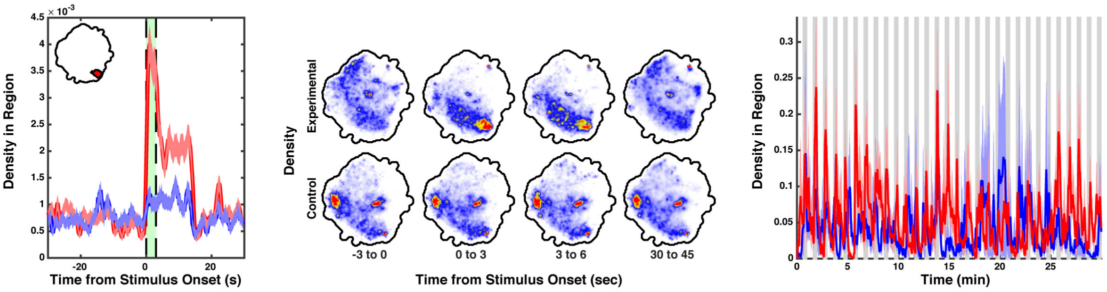

G12

SS02608

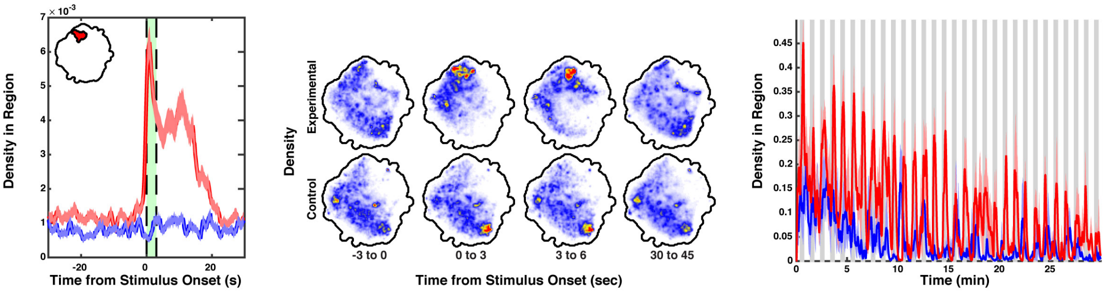

SS02609 (9mW/cM²)

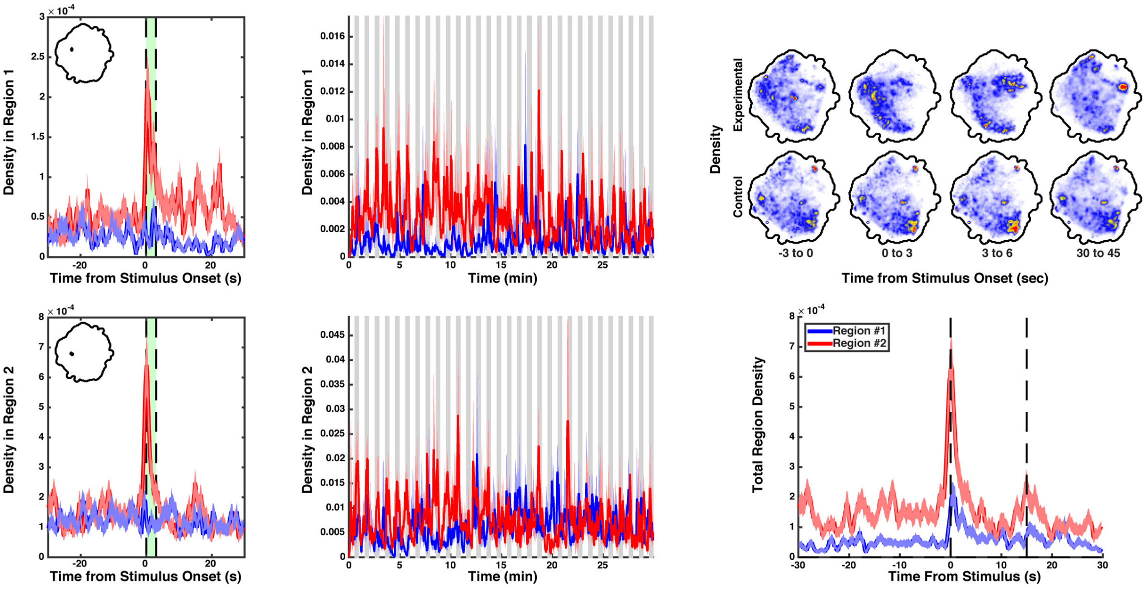

G13

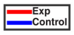

SS01067

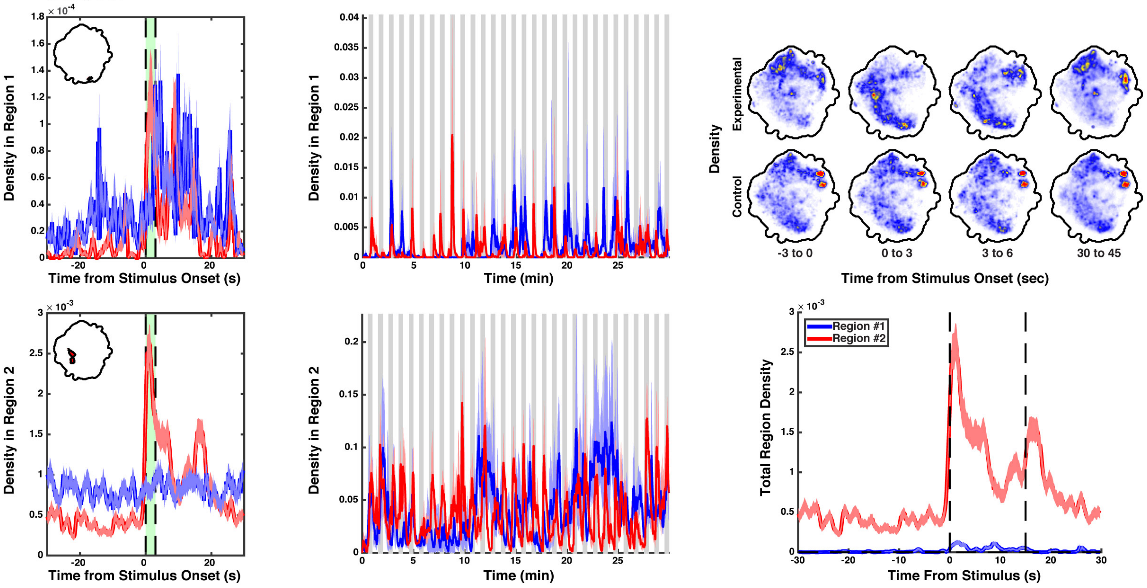

SS02259

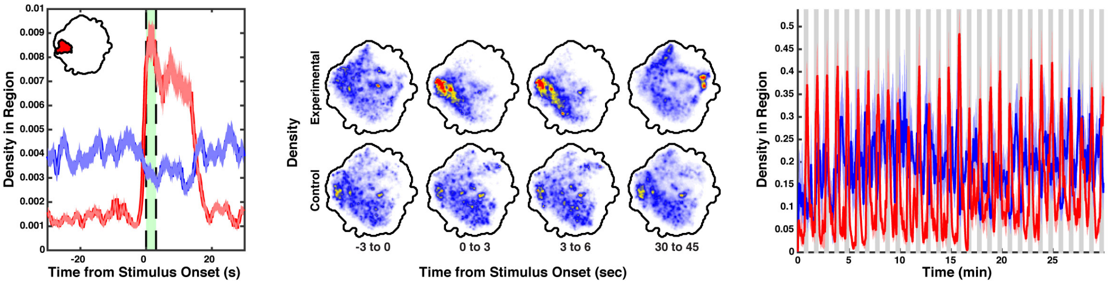

SS01567

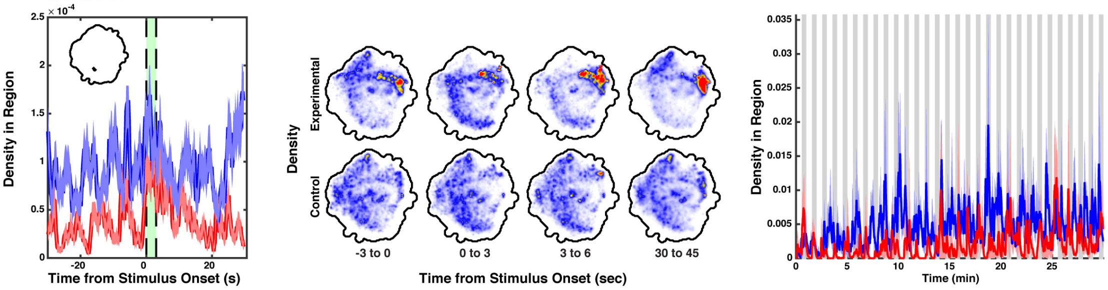

G14

SS01069

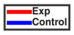

Exp
Control

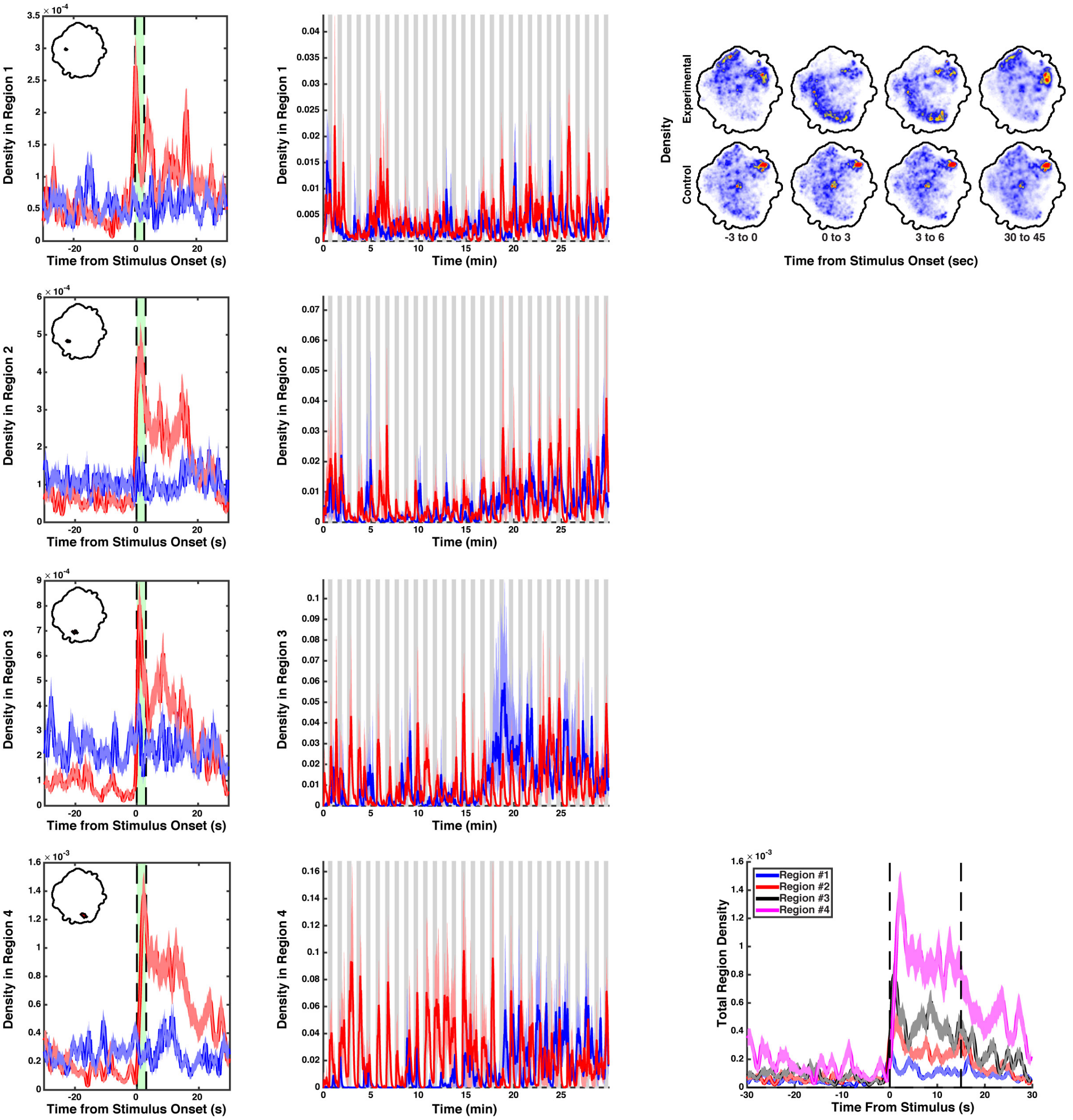

Density in Region 1
Density in Region 2
Density in Region 3
Density in Region 4
Time from Stimulus Onset (s)
Time (min)
Experimental
Control
Density
-3 to 0
0 to 3
3 to 6
30 to 45
Time from Stimulus Onset (sec)
Region #1
Region #2
Region #3
Region #4
Total Region Density
Time From Stimulus (s)

G15

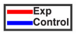

SS02377 (9mW/cM²)

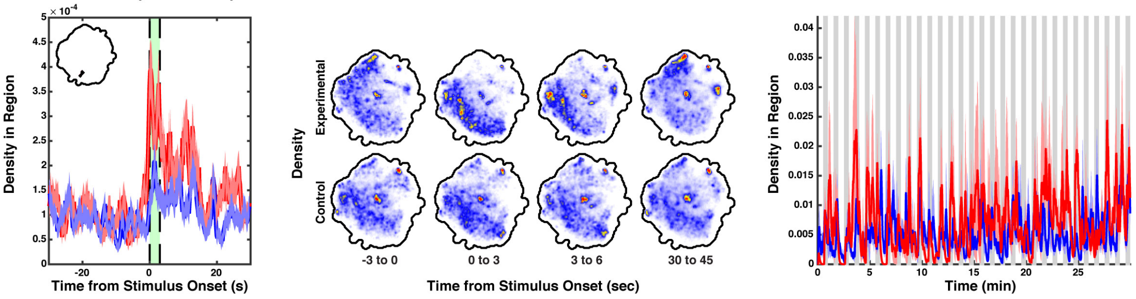

G16

SS01543 (9mW/cM²)

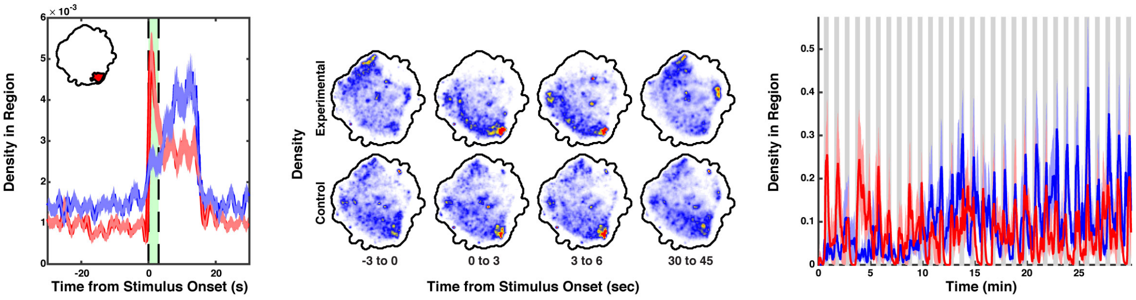

G17

SS00898 (9mW/cM²)

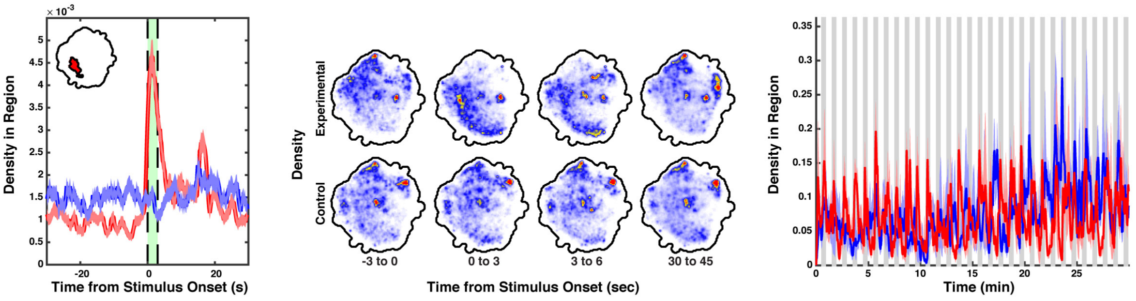

SS02279 (9mW/cM²)

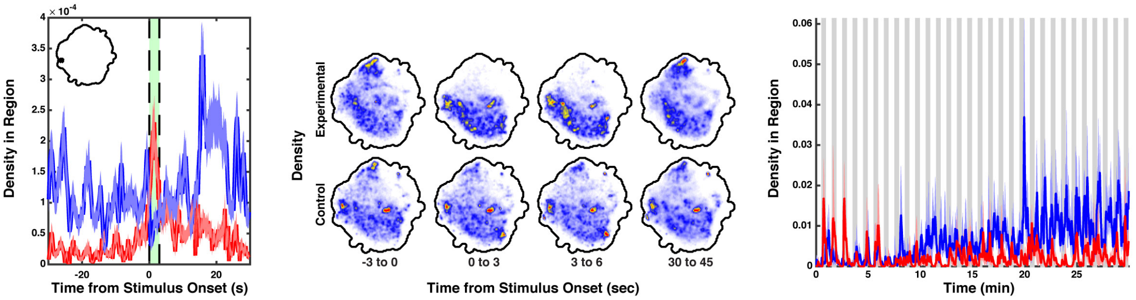

G25

SS01602

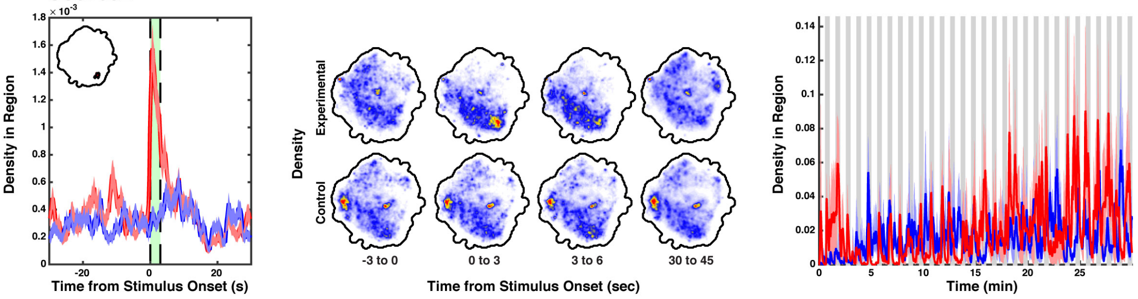

G26

SS01061

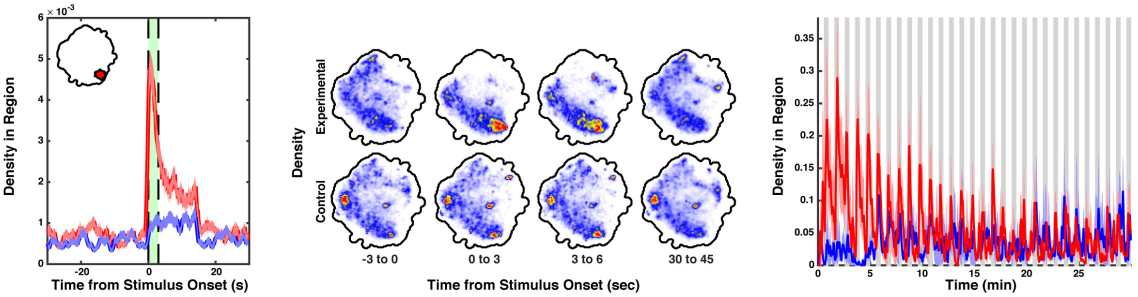

SS01064 (9mW/cM^2)

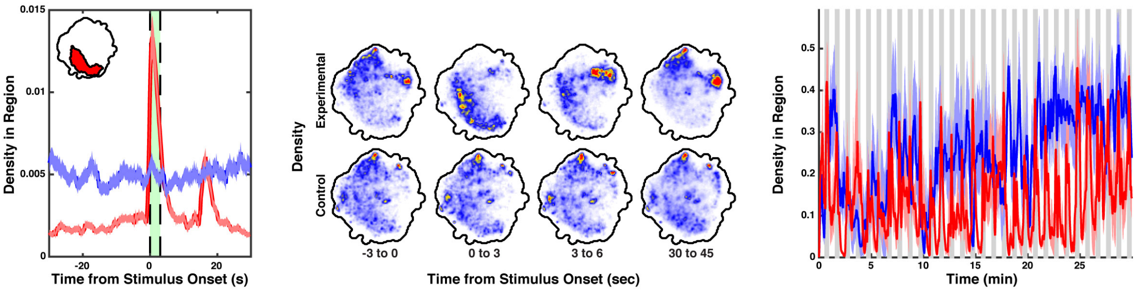

SS01558

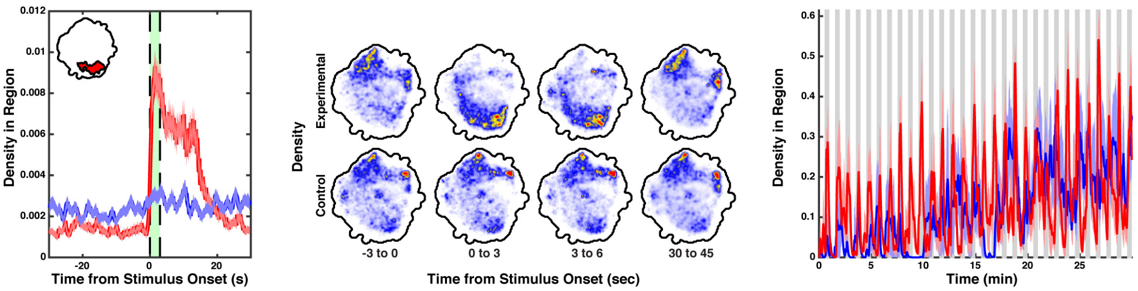

G26

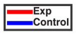

SS01593

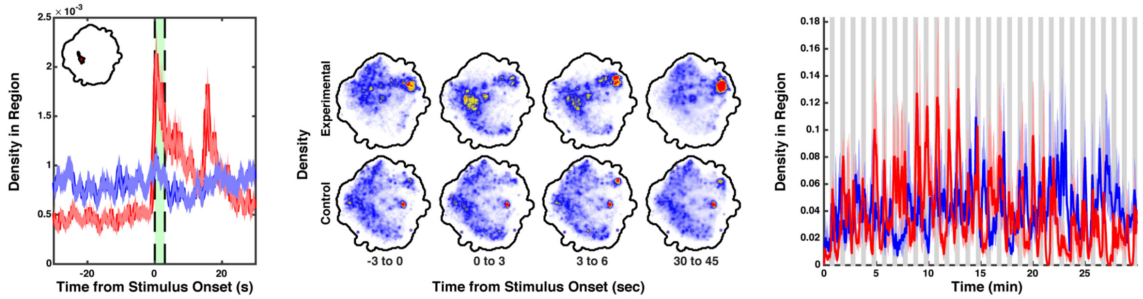

G27

SS01557

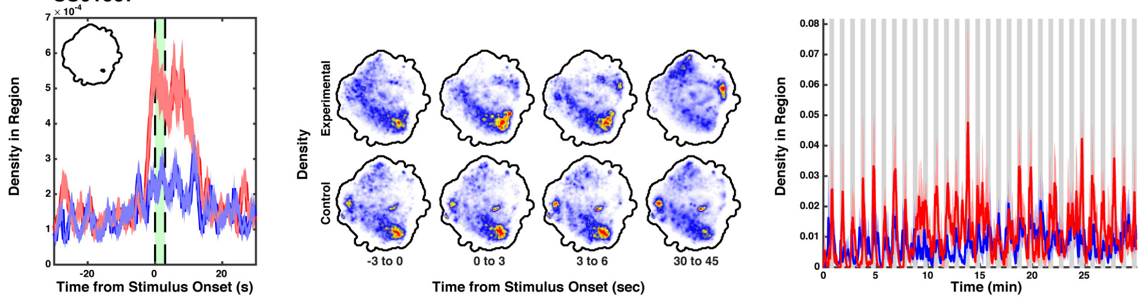

G28

SS01063 (9mW/cM²)

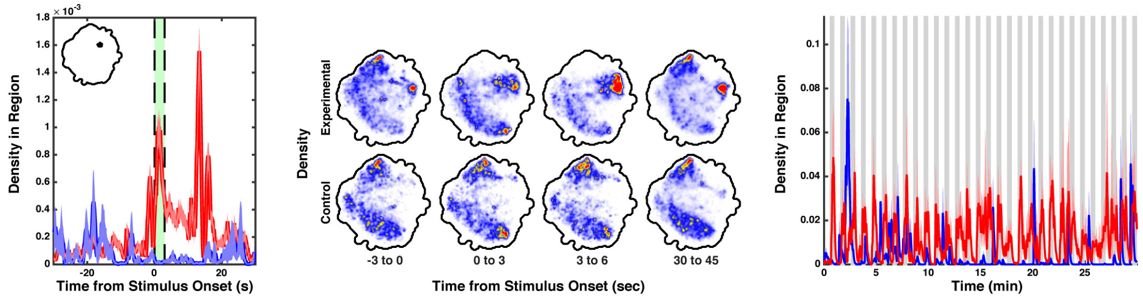

SS01564

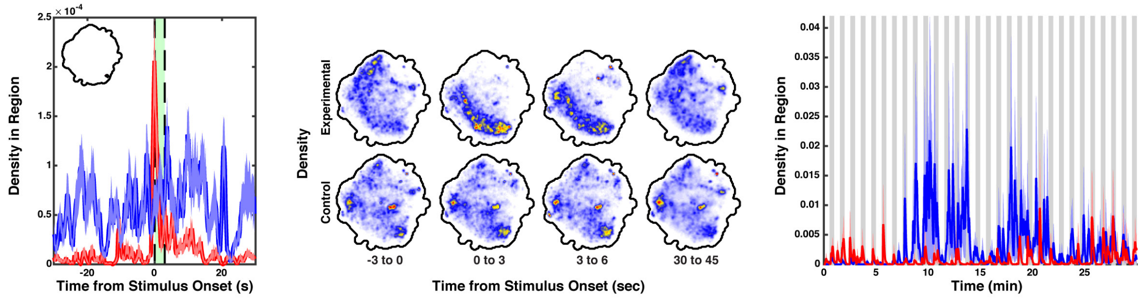

G30

SS01054 (9mW/cM²)

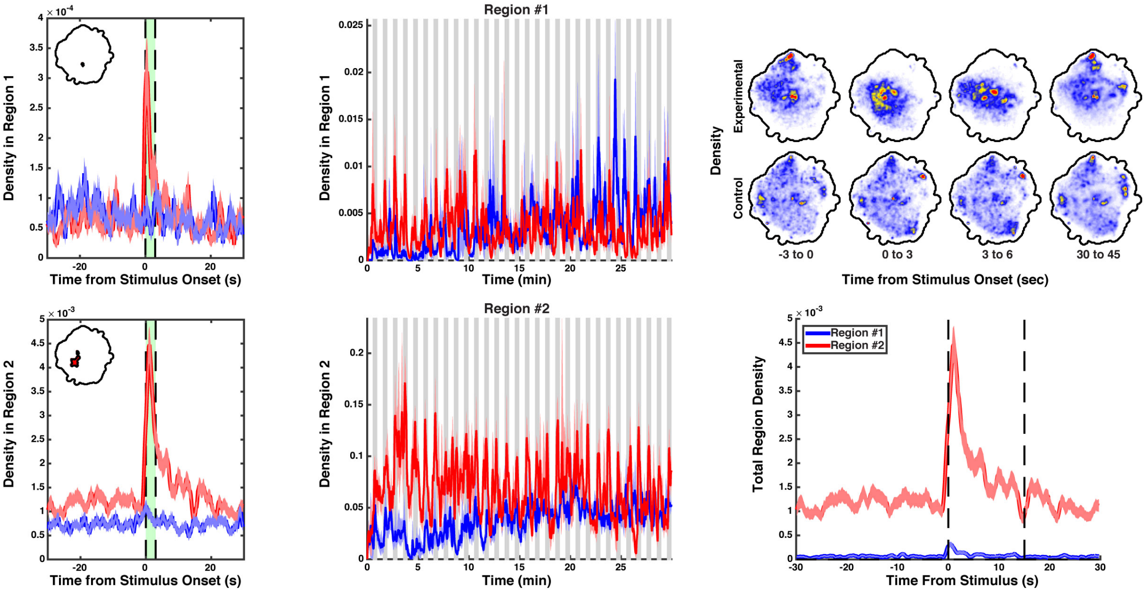

SS01077

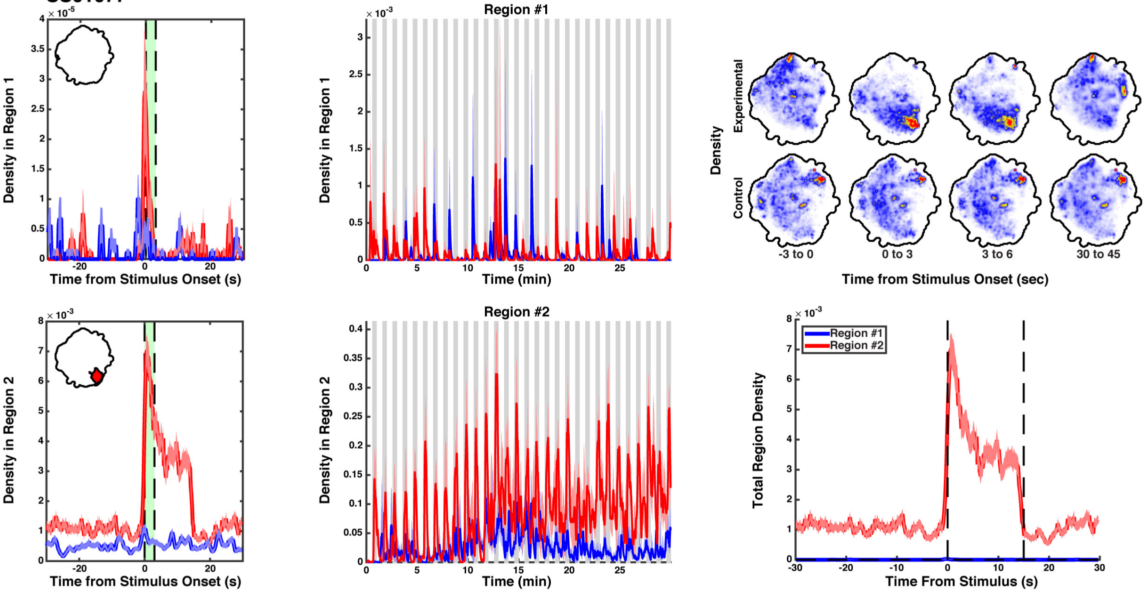

G30

SS02316

SS01588

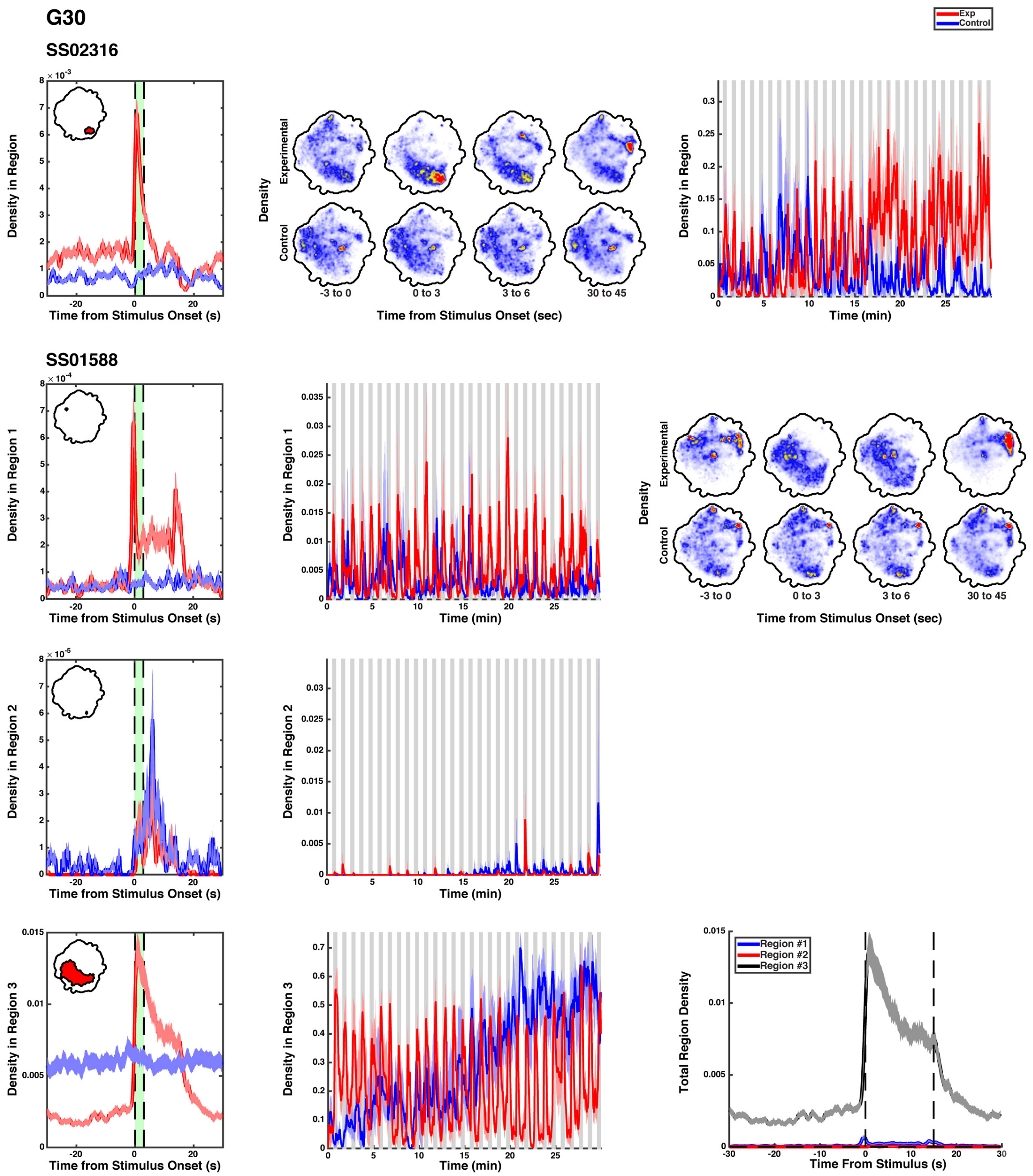

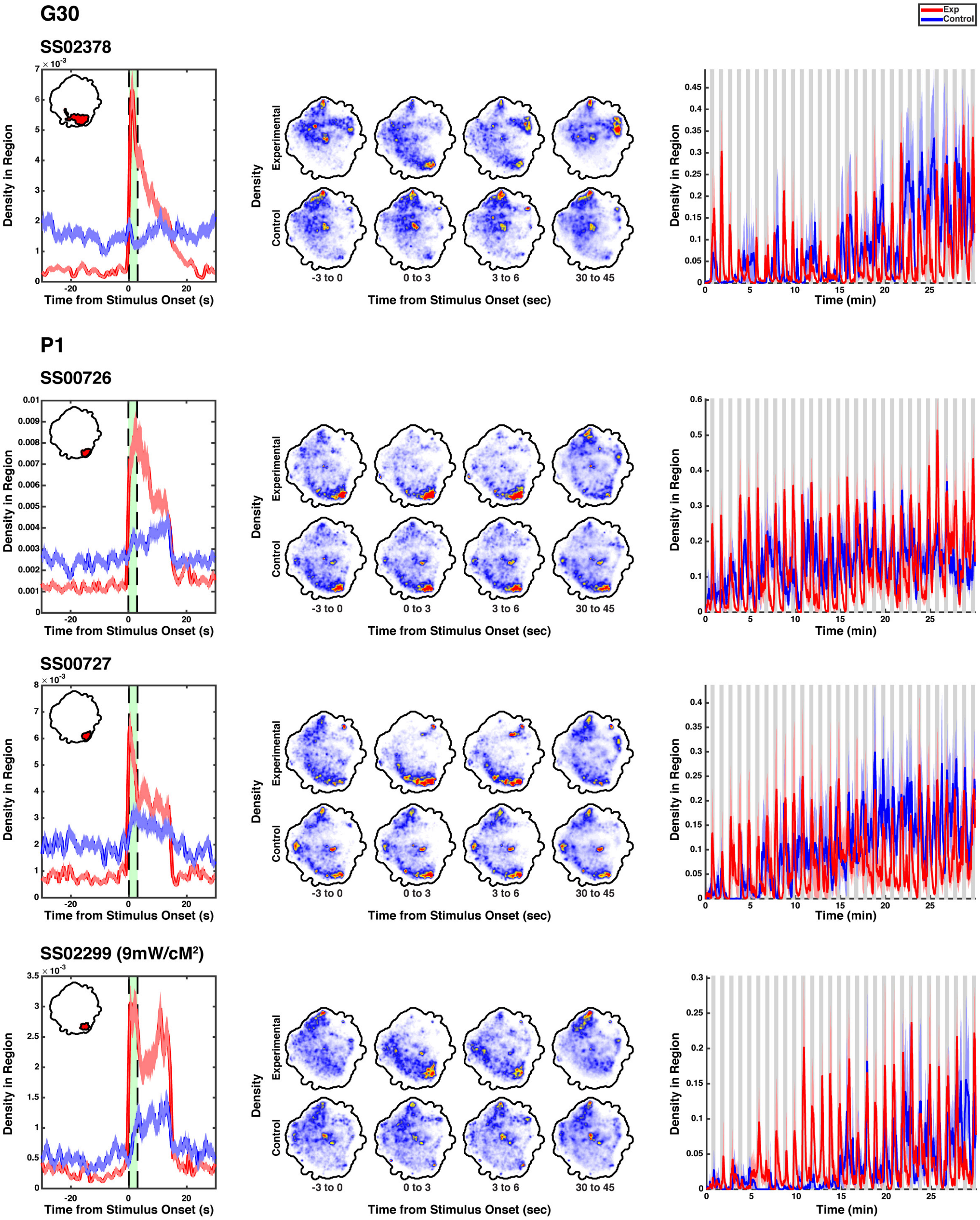
G30
SS02378
Exp
Control
Density in Region
Time from Stimulus Onset (s)
Density
Experimental
Control
-3 to 0
0 to 3
3 to 6
30 to 45
Time from Stimulus Onset (sec)
Time (min)
P1
SS00726
SS00727
SS02299 (9mW/cM²)

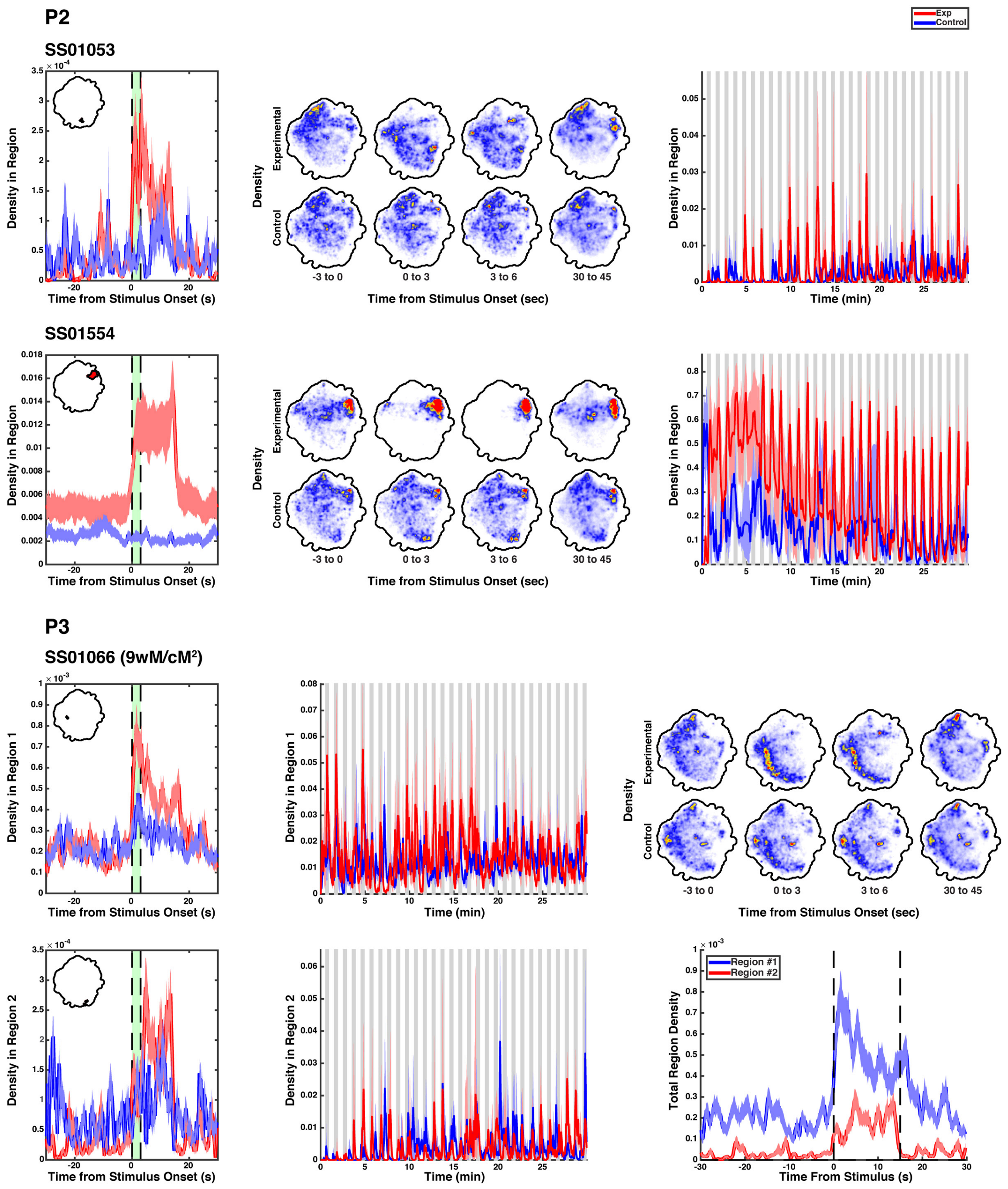
P2
SS01053
Exp
Control
Density in Region
Time from Stimulus Onset (s)
Density
Experimental
Control
-3 to 0
0 to 3
3 to 6
30 to 45
Time from Stimulus Onset (sec)
Time (min)
SS01554
P3
SS01066 (9wM/cM²)
Density in Region 1
Density in Region 2
Region #1
Region #2
Total Region Density
Time From Stimulus (s)

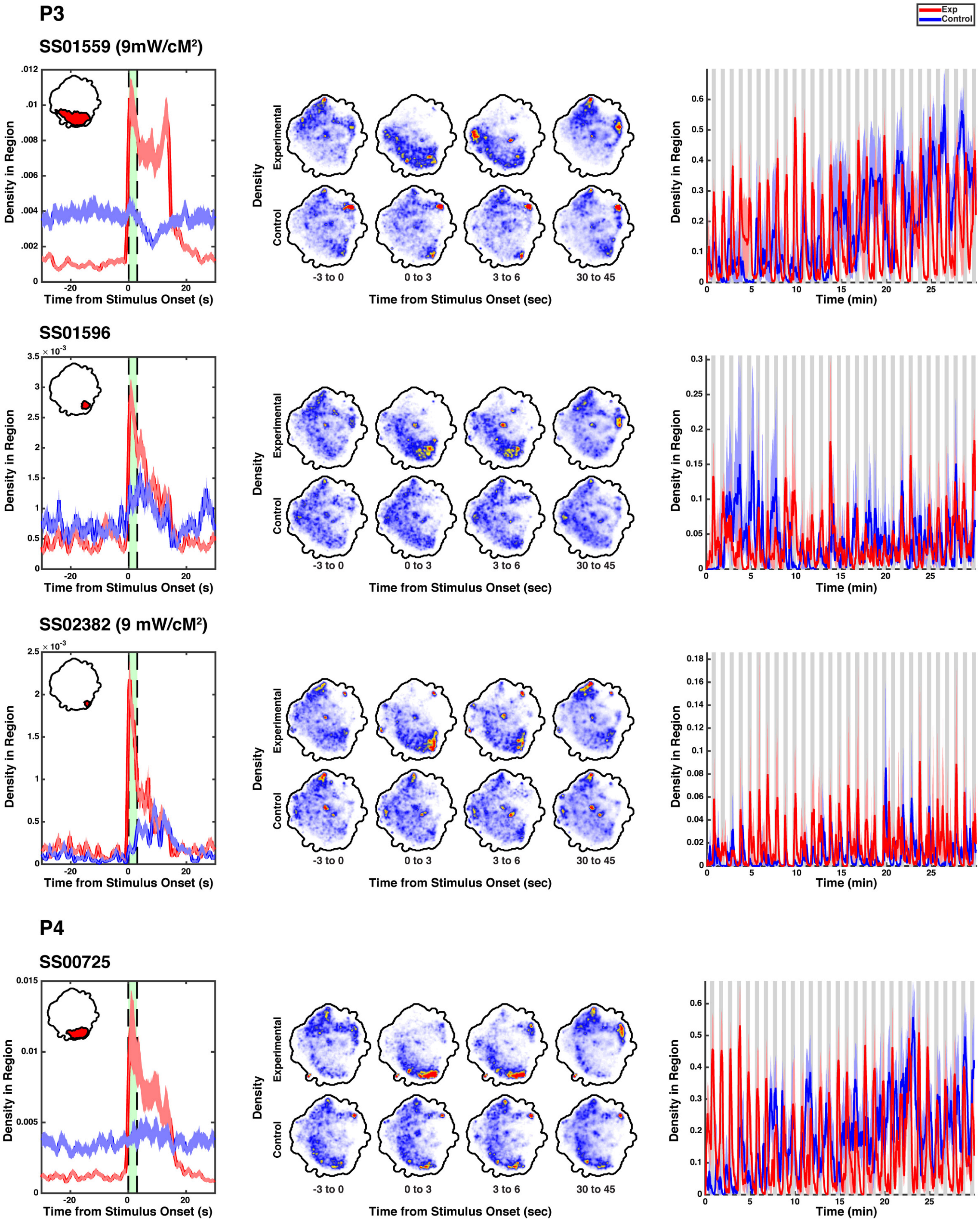
P3
SS01559 (9mW/cM²)
SS01596
SS02382 (9 mW/cM²)
P4
SS00725
Exp
Control
Density in Region
Time from Stimulus Onset (s)
Density
Experimental
Control
-3 to 0
0 to 3
3 to 6
30 to 45
Time from Stimulus Onset (sec)
Time (min)

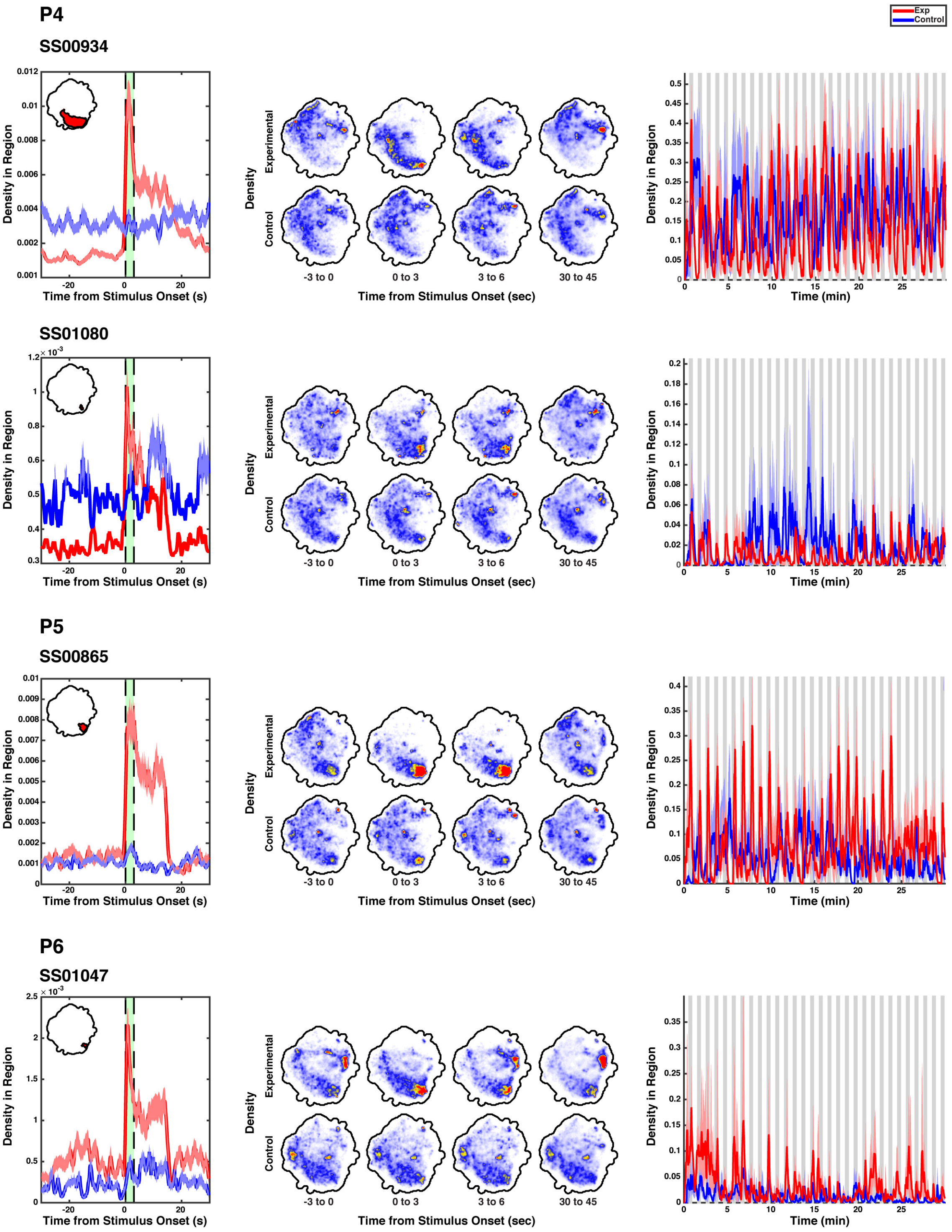
P4
SS00934
SS01080
P5
SS00865
P6
SS01047
Exp
Control
Density in Region
Time from Stimulus Onset (s)
Density
Experimental
Control
-3 to 0
0 to 3
3 to 6
30 to 45
Time from Stimulus Onset (sec)
Time (min)

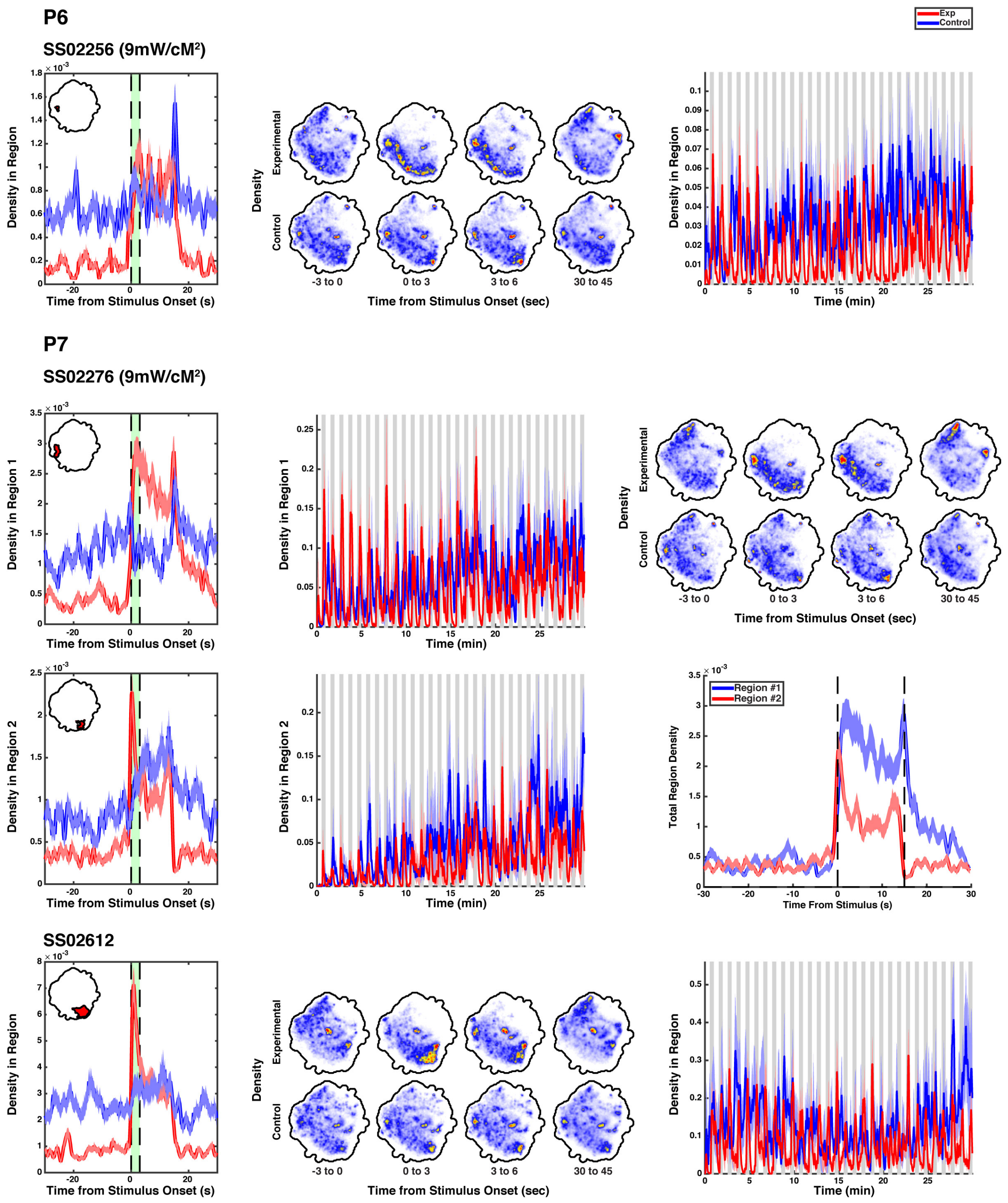

P6
SS02256 (9mW/cM²)
Exp
Control
Density in Region
Time from Stimulus Onset (s)
Density
Experimental
Control
-3 to 0
0 to 3
3 to 6
30 to 45
Time from Stimulus Onset (sec)
Time (min)
P7
SS02276 (9mW/cM²)
Density in Region 1
Density in Region 2
Region #1
Region #2
Total Region Density
Time From Stimulus (s)
SS02612

P9

SS01540

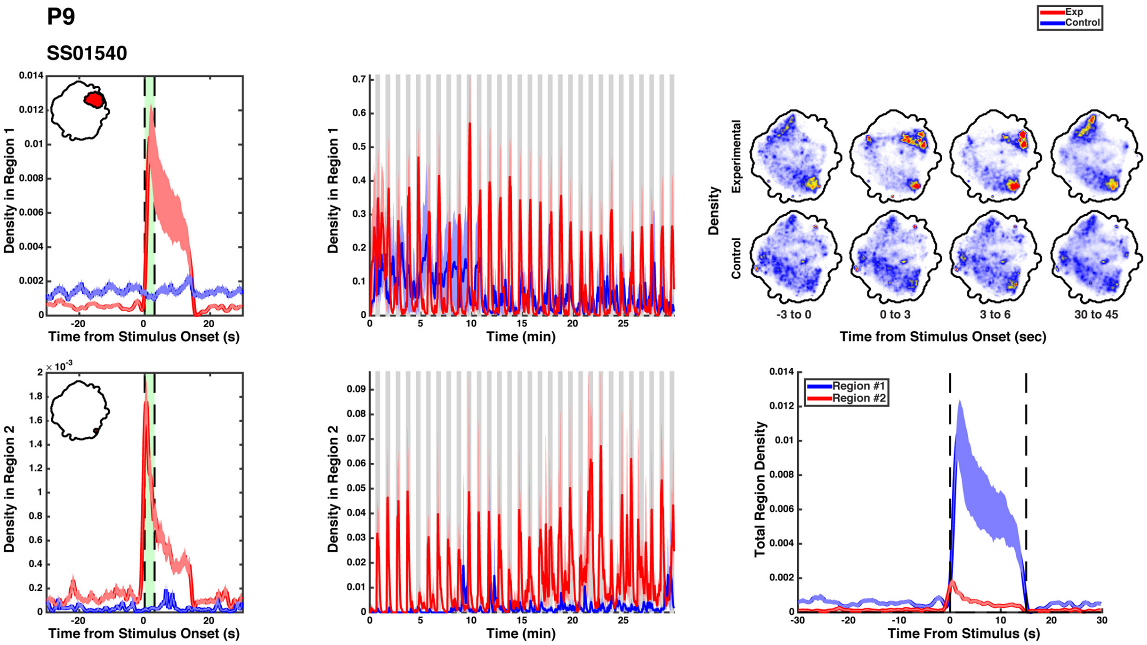

P10

SS01049

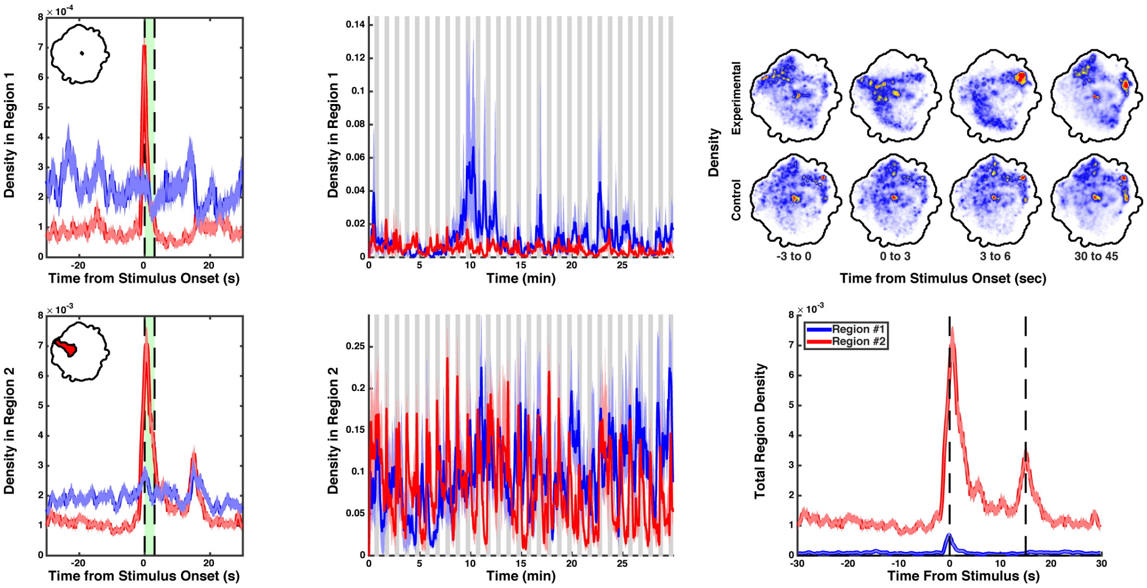

P10

SS01608

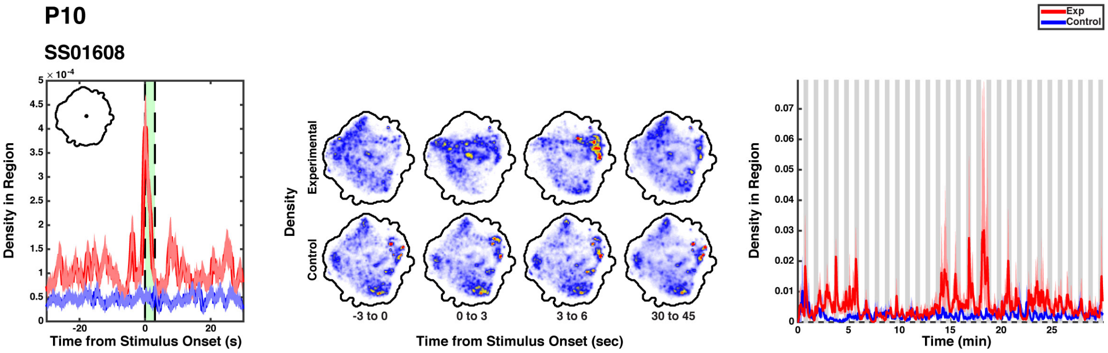

SS02385

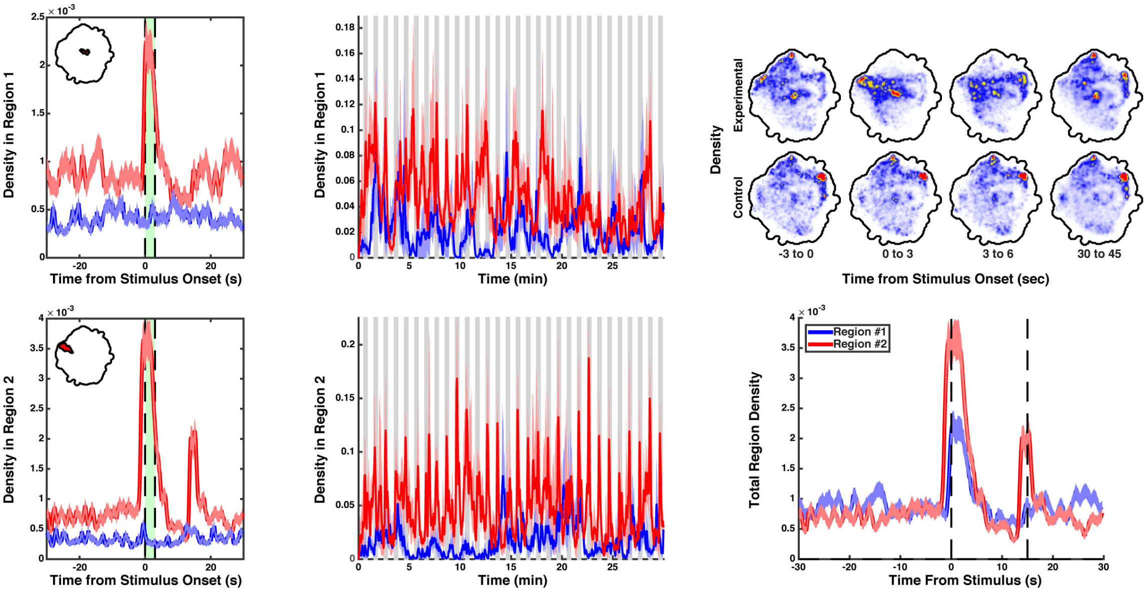

P15

SS01556

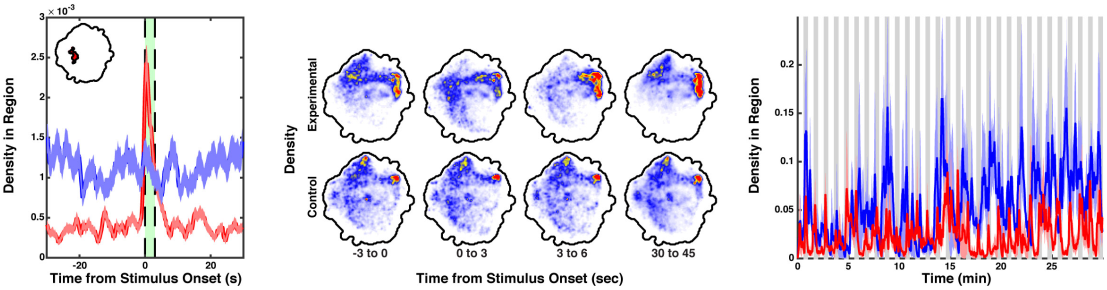

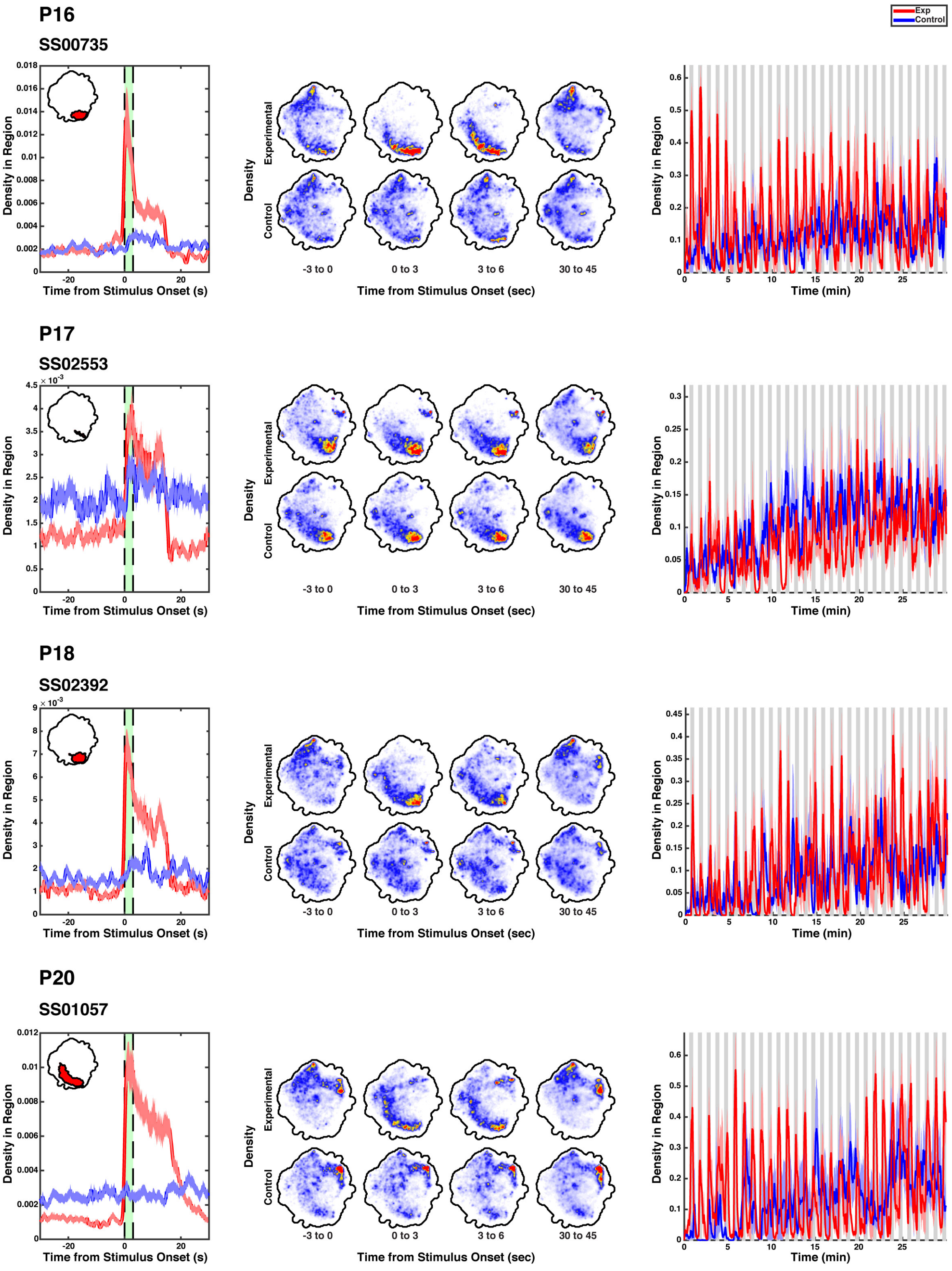

P16
SS00735
P17
SS02553
P18
SS02392
P20
SS01057
Exp
Control
Density in Region
Time from Stimulus Onset (s)
Density
Experimental
Control
-3 to 0
0 to 3
3 to 6
30 to 45
Time from Stimulus Onset (sec)
Time (min)

P20

SS00729 (9mW/cM^2)

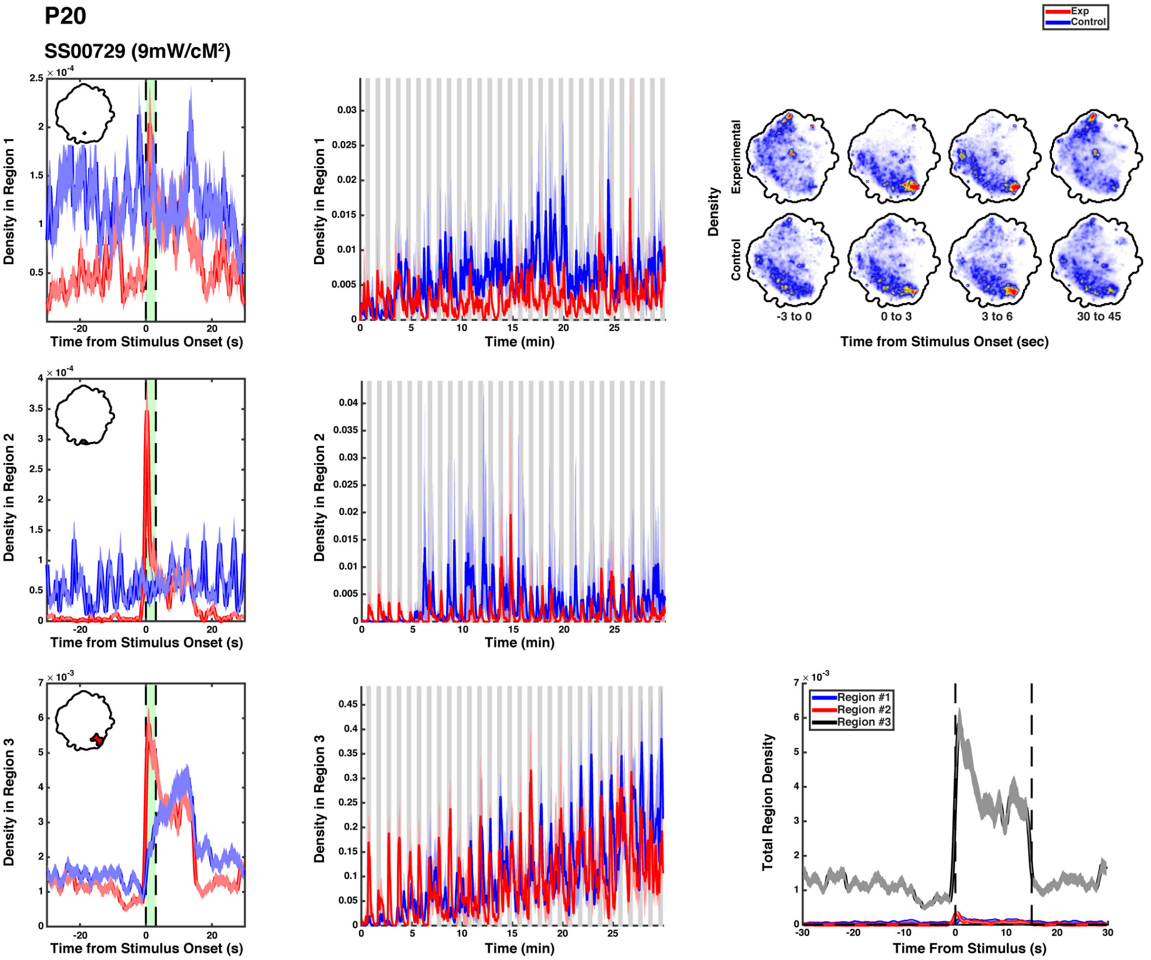

SS01078 (9mW/cM^2)

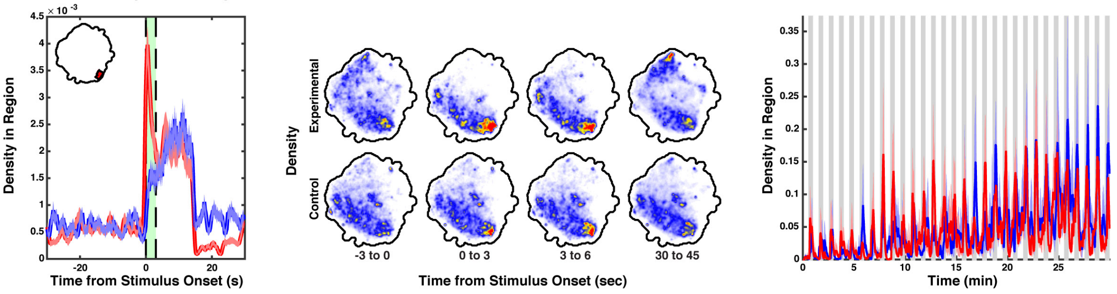

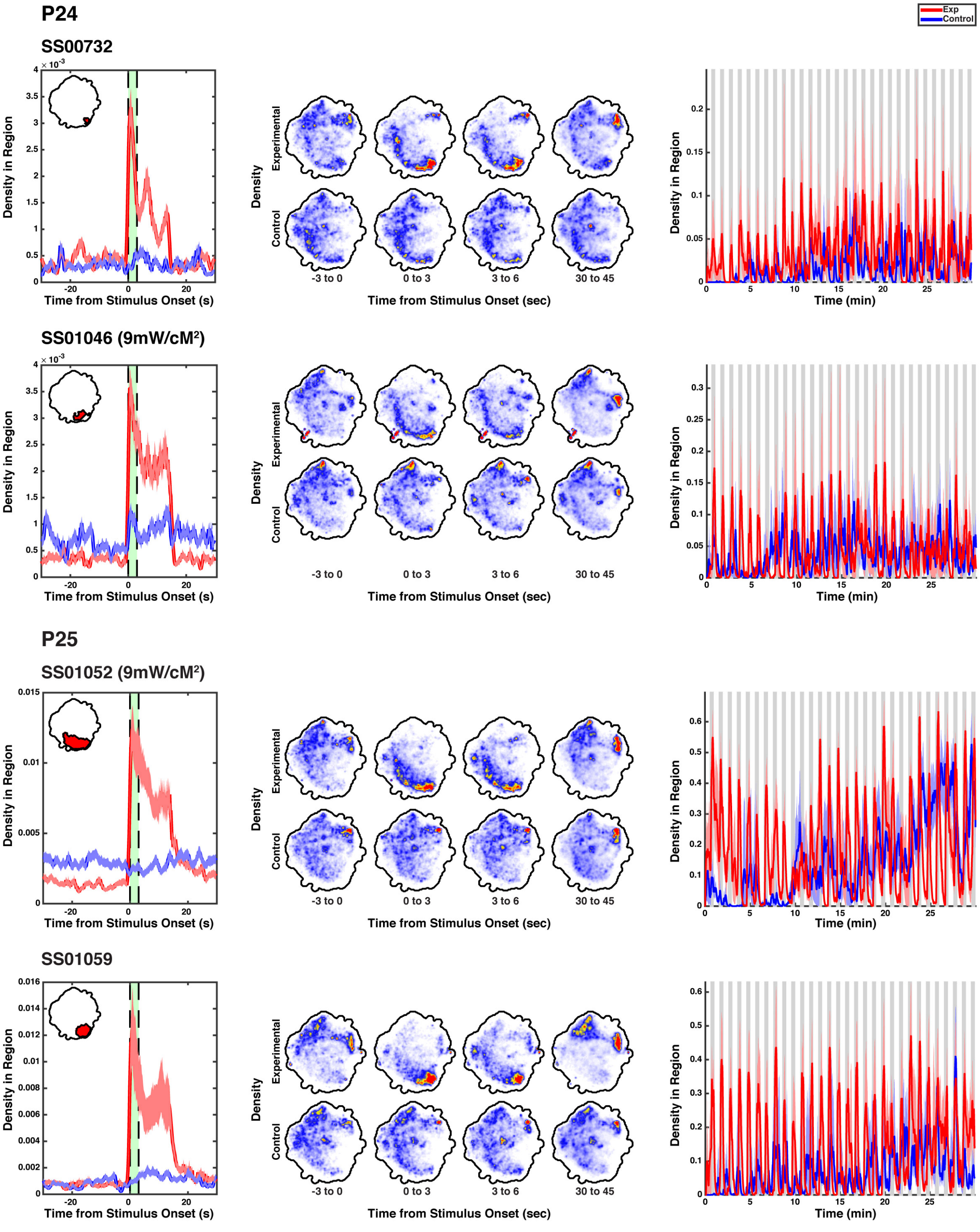
P24
SS00732
Exp
Control
Density in Region
Time from Stimulus Onset (s)
Experimental
Control
Density
-3 to 0
0 to 3
3 to 6
30 to 45
Time from Stimulus Onset (sec)
Density in Region
Time (min)
SS01046 (9mW/cM²)
Density in Region
Time from Stimulus Onset (s)
Experimental
Control
Density
-3 to 0
0 to 3
3 to 6
30 to 45
Time from Stimulus Onset (sec)
Density in Region
Time (min)
P25
SS01052 (9mW/cM²)
Density in Region
Time from Stimulus Onset (s)
Experimental
Control
Density
-3 to 0
0 to 3
3 to 6
30 to 45
Time from Stimulus Onset (sec)
Density in Region
Time (min)
SS01059
Density in Region
Time from Stimulus Onset (s)
Experimental
Control
Density
-3 to 0
0 to 3
3 to 6
30 to 45
Time from Stimulus Onset (sec)
Density in Region
Time (min)

P26

SS01553

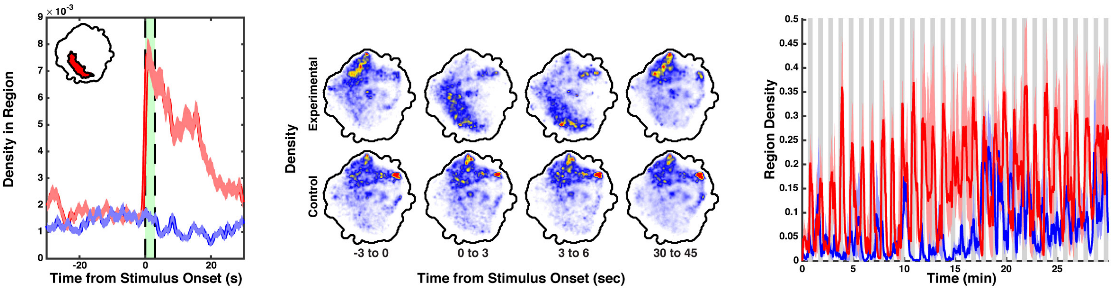

P27

SS02257 (9mW/cM²)

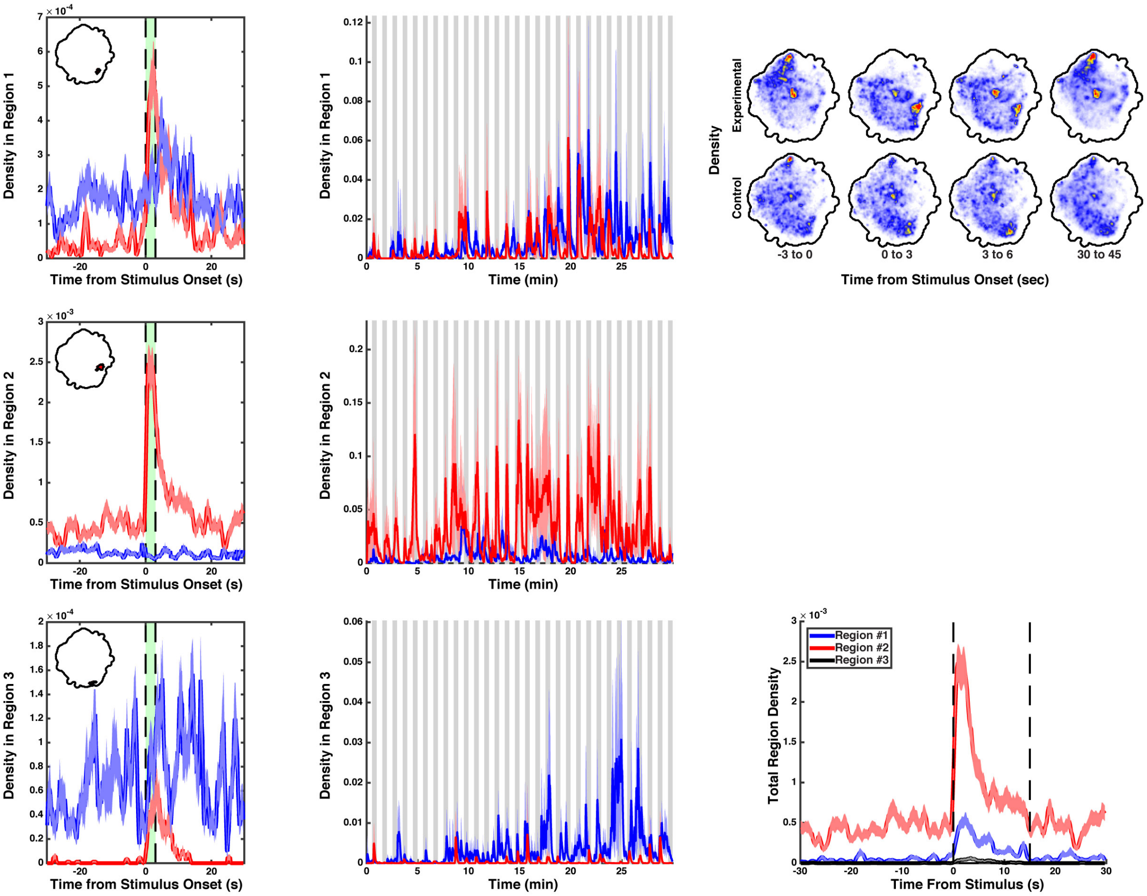

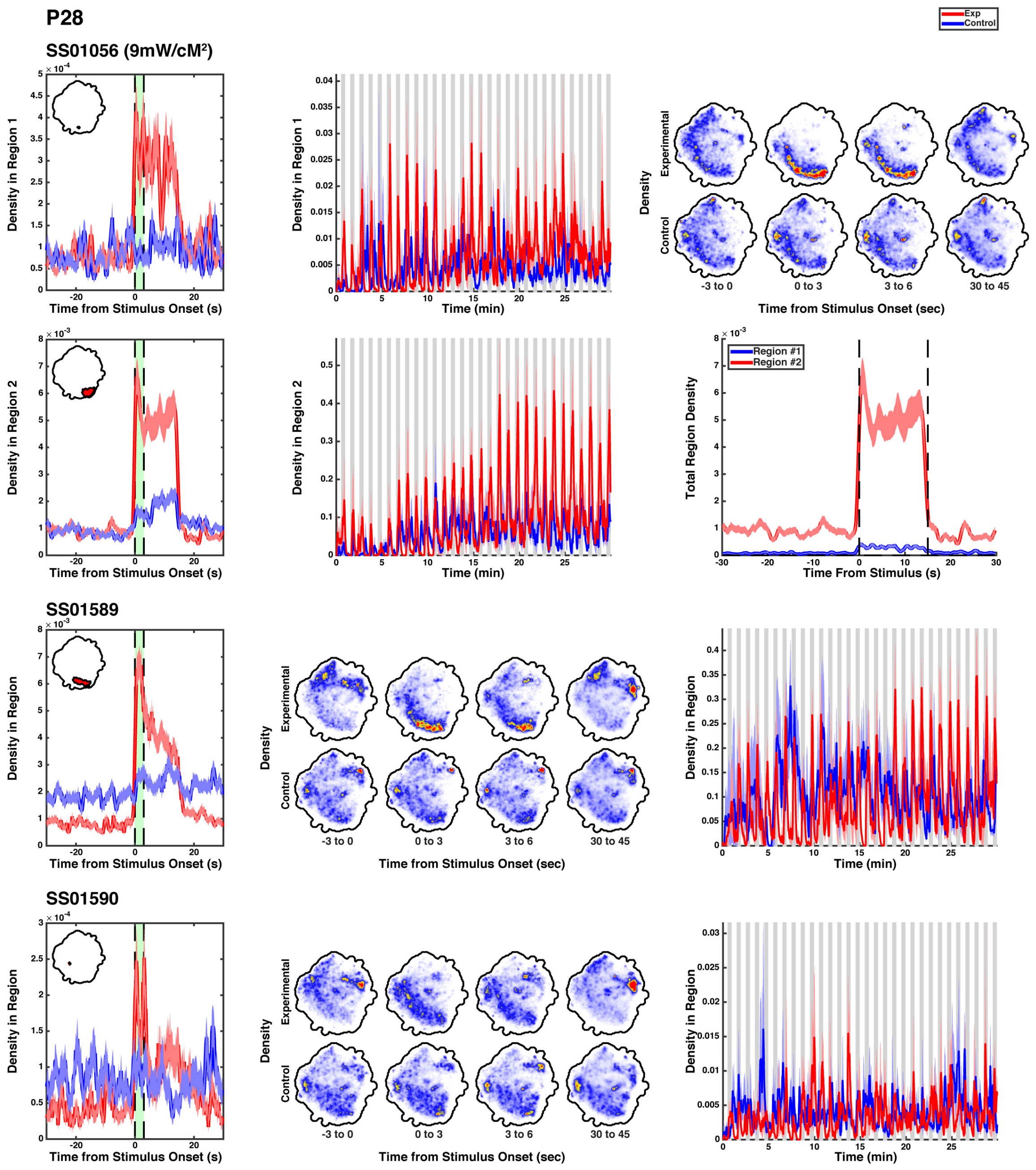
P28
Exp
Control
SS01056 (9mW/cM²)
Density in Region 1
Time from Stimulus Onset (s)
Time (min)
Experimental
Control
Density
-3 to 0
0 to 3
3 to 6
30 to 45
Time from Stimulus Onset (sec)
Density in Region 2
Region #1
Region #2
Total Region Density
Time From Stimulus (s)
SS01589
Density in Region
SS01590

P29

SS01587

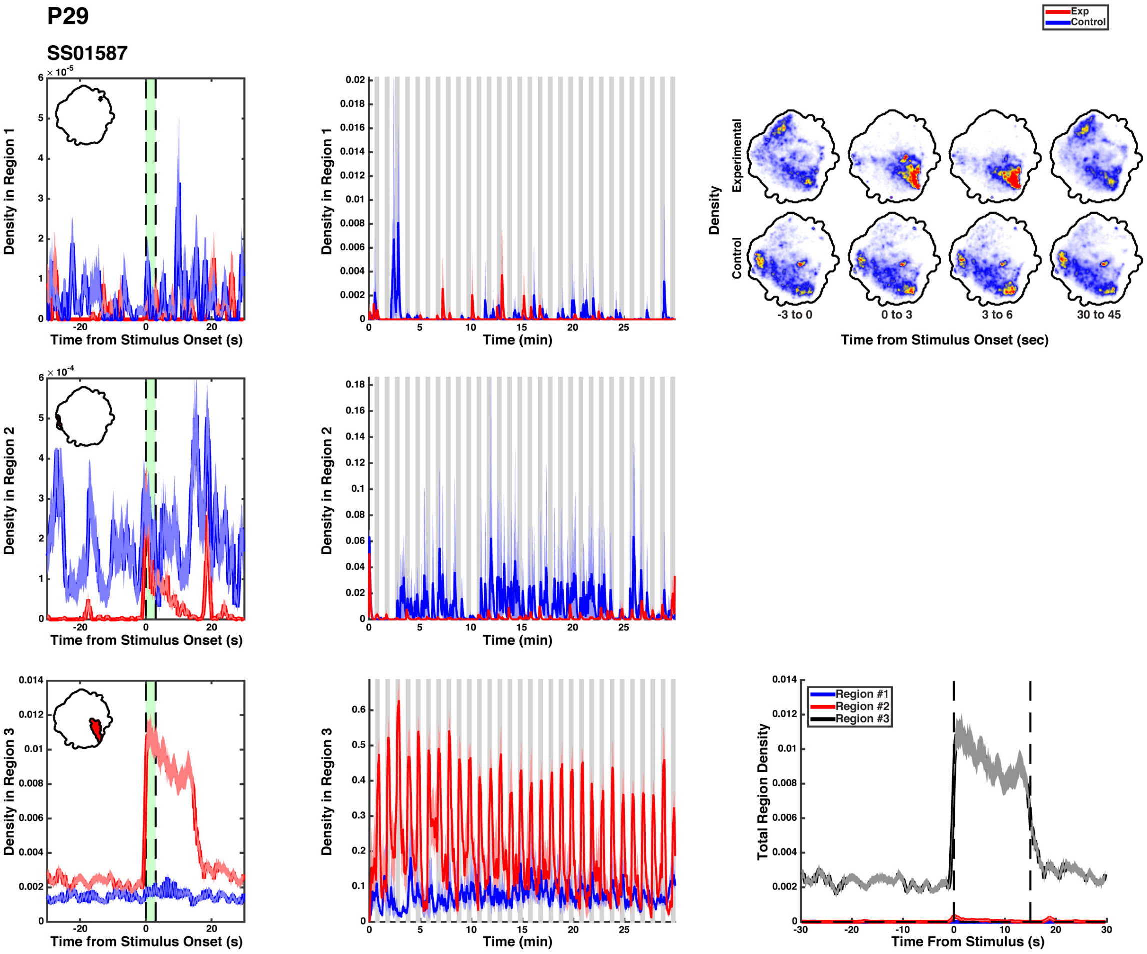

Exp
Control
Density in Region 1
Time from Stimulus Onset (s)
Time (min)
Density
Experimental
Control
-3 to 0
0 to 3
3 to 6
30 to 45
Time from Stimulus Onset (sec)
Density in Region 2
Density in Region 3
Region #1
Region #2
Region #3
Total Region Density
Time From Stimulus (s)

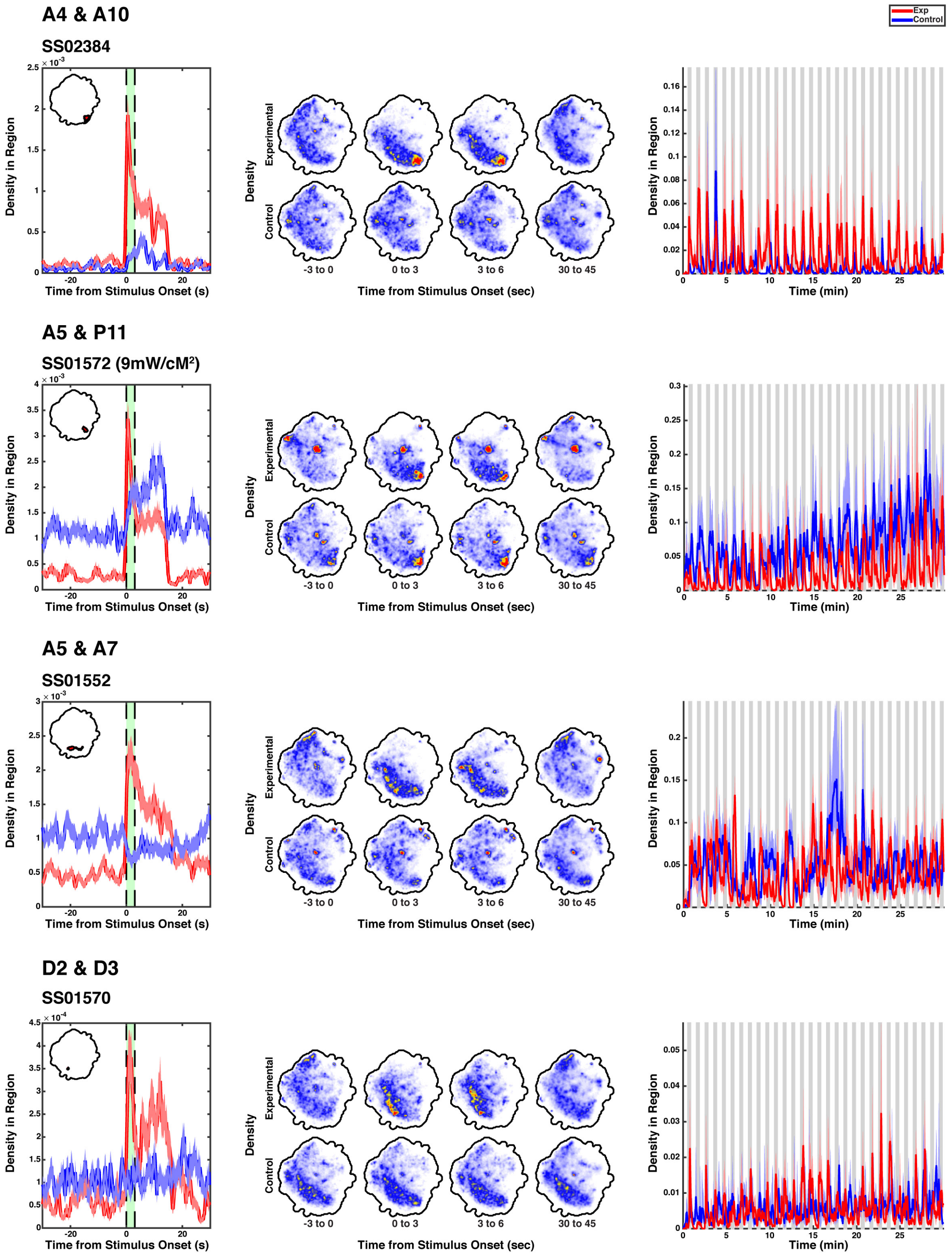
A4 & A10
SS02384
Exp
Control
Density in Region
Time from Stimulus Onset (s)
Experimental
Control
Density
-3 to 0
0 to 3
3 to 6
30 to 45
Time from Stimulus Onset (sec)
Time (min)
A5 & P11
SS01572 (9mW/cM²)
A5 & A7
SS01552
D2 & D3
SS01570

D2 & D3

SS01569

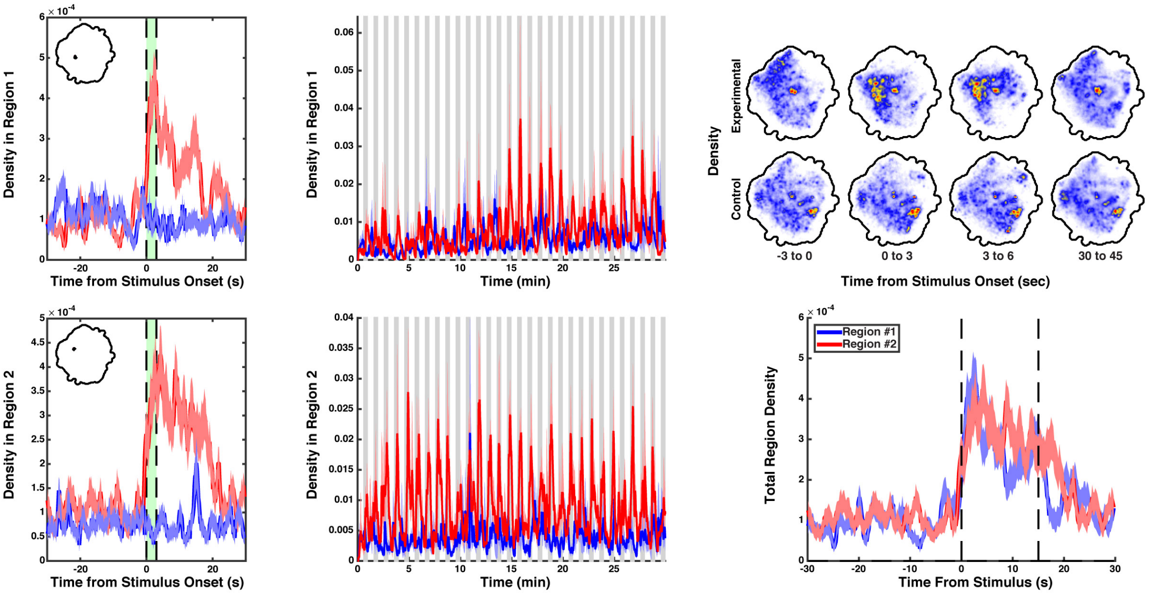

SS01576 (9mW/cM²)

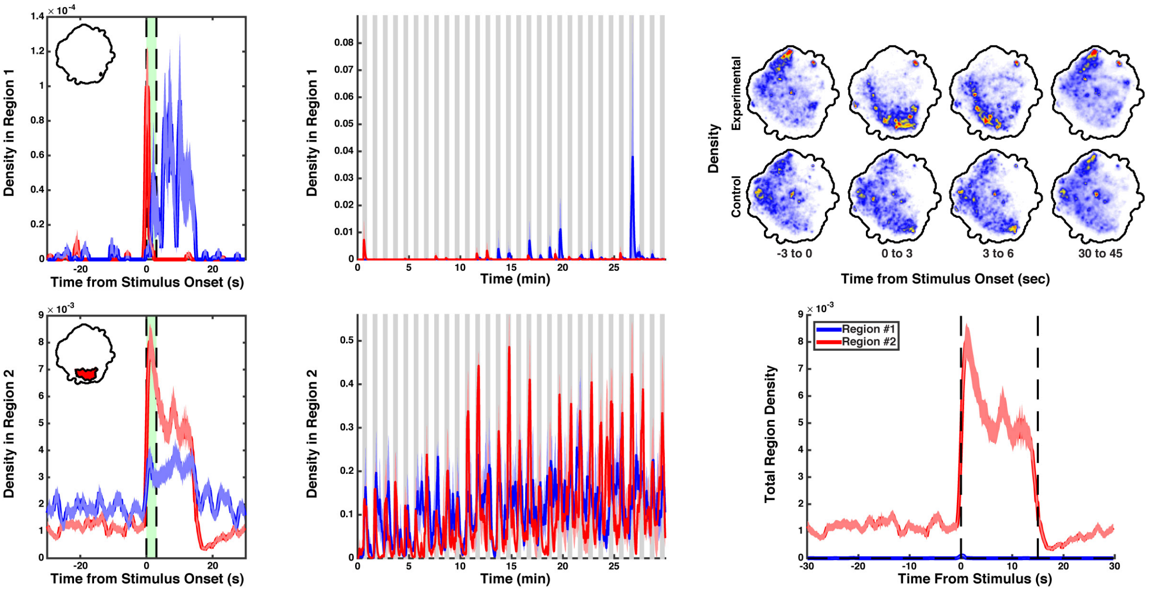

D2 & D3

SS01068 (9mW/cM²)

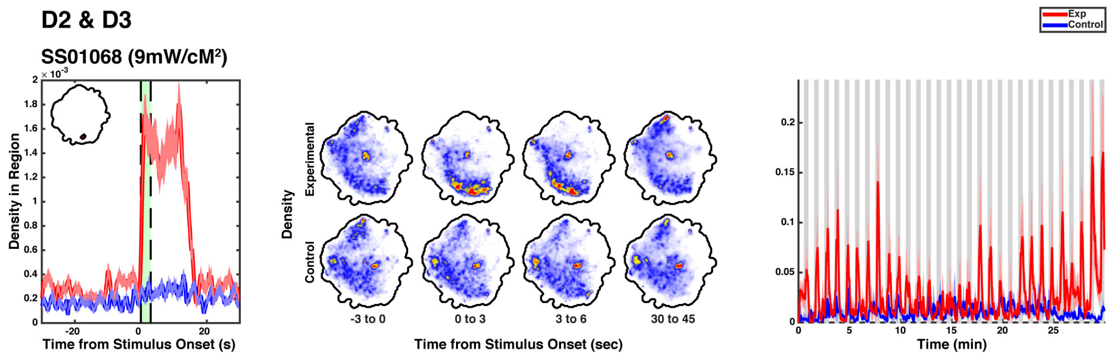

P2 & P11

SS02379 (9mW/cM²)

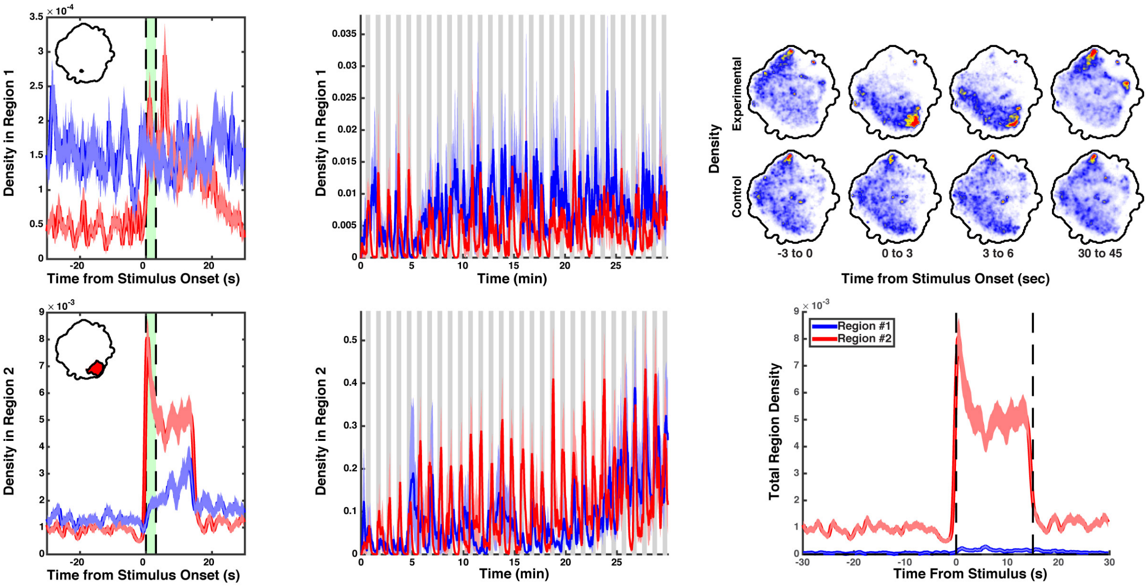

SS02534 (9mW/cM²)

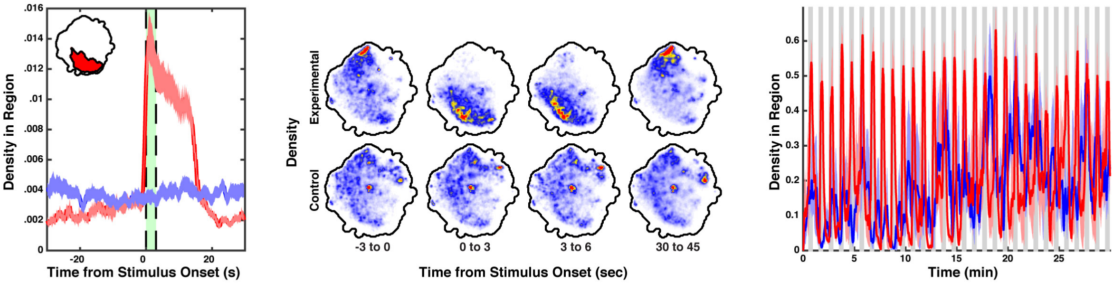

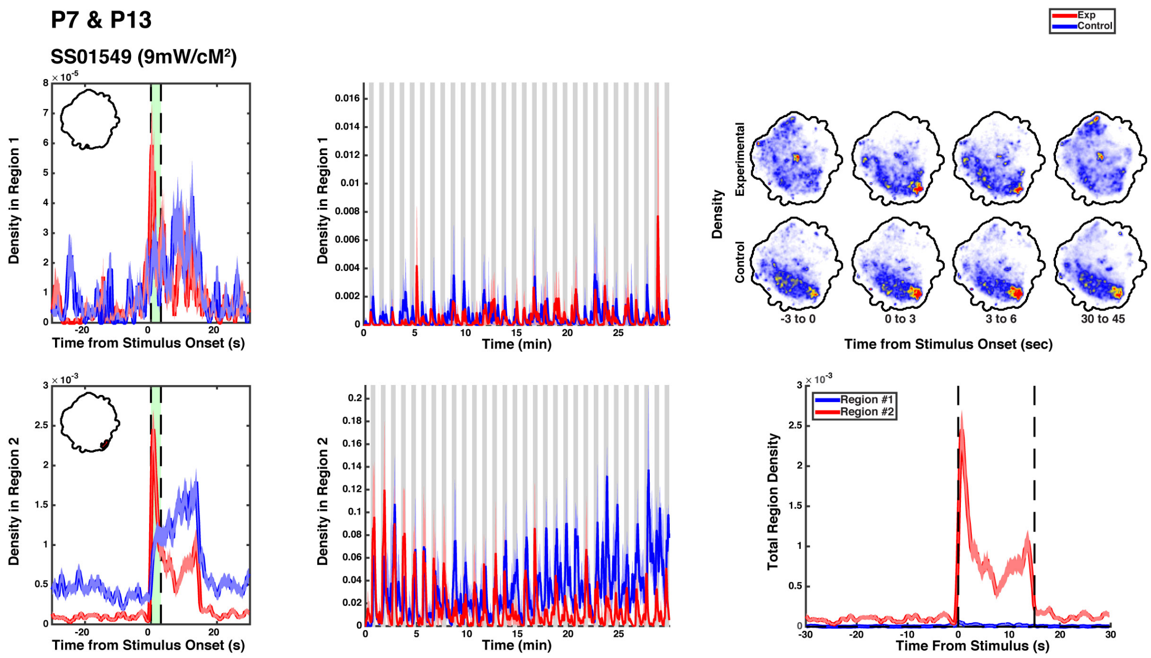

Figure S5. Analysis of all the descending neuron split GAL4 lines. The line by line analysis shown here follows the model of the selected examples described in Figure 2 of the main text.
